# Surrogate Adiposity Markers and Mortality

**DOI:** 10.1001/jamanetworkopen.2023.34836

**Published:** 2023-09-20

**Authors:** Irfan Khan, Michael Chong, Ann Le, Pedrum Mohammadi-Shemirani, Robert Morton, Christina Brinza, Michel Kiflen, Sukrit Narula, Loubna Akhabir, Shihong Mao, Katherine Morrison, Marie Pigeyre, Guillaume Paré

**Affiliations:** 1 Population Health Research Institute, David Braley Cardiac, Vascular and Stroke Research Institute, Hamilton, Ontario, Canada; 2 Thrombosis and Atherosclerosis Research Institute, David Barley Cardiac, Vascular and Stroke Research, Hamilton, Ontario, Canada; 3 Department of Pathology and Molecular Medicine, Michael G. DeGroote School of Medicine, McMaster University, Hamilton, Ontario, Canada; 4 Department of Health Research Methods, Evidence, and Impact, McMaster University, Hamilton, Ontario, Canada; 5 College of Medicine and Health, University College Cork, Cork, Ireland; 6 School of Medicine, Queen’s University, Kingston, Ontario, Canada; 7 Temerty Faculty of Medicine, University of Toronto, Medical Sciences Building, Toronto, Ontario, Canada; 8 Department of Internal Medicine, Yale University School of Medicine, New Haven, Connecticut; 9 Department of Pediatrics, McMaster University, Hamilton, Ontario, Canada; 10 Centre for Metabolism, Obesity and Diabetes Research, McMaster University, Hamilton, Ontario, Canada

## Abstract

**Importance:**

Body mass index (BMI) is an easily obtained adiposity surrogate. However, there is variability in body composition and adipose tissue distribution between individuals with the same BMI, and there is controversy regarding the BMI associated with the lowest mortality risk.

**Objective:**

To evaluate which of BMI, fat mass index (FMI), and waist-to-hip (WHR) has the strongest and most consistent association with mortality.

**Design, Setting, and Participant:**

This cohort study used incident deaths from the UK Biobank (UKB; 2006-2022), which includes data from 22 clinical assessment centers across the United Kingdom. UKB British participants of British White ancestry (N = 387 672) were partitioned into a discovery cohort (n = 337 078) and validation cohort (n = 50 594), with the latter consisting of 25 297 deaths and 25 297 controls. The discovery cohort was used to derive genetically determined adiposity measures while the validation cohort was used for analyses. Exposure-outcome associations were analyzed through observational and mendelian randomization (MR) analyses.

**Exposures:**

BMI, FMI, and WHR.

**Main Outcomes and Measures:**

All-cause and cause-specific (cancer, cardiovascular disease [CVD], respiratory disease, or other causes) mortality.

**Results:**

There were 387 672 and 50 594 participants in our observational (mean [SD] age, 56.9 [8.0] years; 177 340 [45.9%] male, 210 332 [54.2%], female), and MR (mean [SD] age, 61.6 [6.2] years; 30 031 [59.3%] male, 20 563 [40.6%], female) analyses, respectively. Associations between measured BMI and FMI with all-cause mortality were J-shaped, whereas the association of WHR with all-cause mortality was linear using the hazard ratio (HR) scale (HR per SD increase of WHR, 1.41 [95% CI, 1.38-1.43]). Genetically determined WHR had a stronger association with all-cause mortality than BMI (odds ratio [OR] per SD increase of WHR, 1.51 [95% CI, 1.32-1.72]; OR per SD increase of BMI, 1.29 [95% CI, 1.20-1.38]; *P *for heterogeneity = .02). This association was stronger in male than female participants (OR, 1.89 [95% CI, 1.54-2.32]; *P *for heterogeneity = .01). Unlike BMI or FMI, the genetically determined WHR–all-cause mortality association was consistent irrespective of observed BMI.

**Conclusions and Relevance:**

In this cohort study, WHR had the strongest and most consistent association with mortality irrespective of BMI. Clinical recommendations should consider focusing on adiposity distribution compared with mass.

## Key Points

**Question:**

Among body mass index, fat mass index, and waist-to-hip (WHR) ratio, what is the optimal adiposity measure with the strongest association with mortality outcomes in adults?

**Findings:**

In this cohort study consisting of 387 672 UK adult participants from the UK Biobank, WHR was found to have the strongest and most consistent association with all-cause and cause-specific mortality.

**Meaning:**

In this study, WHR had the most robust association with mortality risk and may serve as a more appropriate target for health care intervention.

## Teaser

This cohort study evaluates which of body mass index (BMI), fat mass index (FMI), and waist-to-hip ratio (WHR) has the strongest and most consistent association with mortality.

## Introduction

Since the 1980s, the global prevalence of individuals with overweight or obesity has doubled to nearly one-third of the world’s population.^1^ Current recommendations for treating obesity are largely based on body mass index (BMI; calculated as weight in kilograms divided by height in meters squared). The World Health Organization (WHO) defines a healthy BMI between 18.5 and 24.5 based on observational studies that found these individuals had the lowest disease or mortality risk.^2,3,4^ Previous literature has shown that BMI and all-cause mortality share a J-shaped association, with individuals having a higher risk of mortality outside of the WHO normal range.^3^ However, in recent studies, the BMI range associated with the lowest risk of mortality varies depending on secular trends, ethnicity, and population.^5,6,7,8^ Thus, BMI may not be the best clinical adiposity measure, as higher BMI could either be beneficial or deleterious depending on clinical context. Other adiposity measures that account for body composition and fat distribution, such as fat mass index (FMI) or waist-to-hip ratio (WHR), have been shown to be superior to BMI in their association with disease or mortality risk in observational studies.^9,10,11,12^

Much of the literature regarding the adiposity-mortality association is observational in design. Therefore, these studies are subject to potential residual confounding and reverse causality biases, limiting causal inferences.^9^ It is thus unclear whether BMI is the best clinical adiposity measure in terms of its association with disease or mortality.^12^ Mendelian randomization (MR) uses genetic variants to infer causal relationships between an exposure (eg, BMI) and an outcome (eg, all-cause mortality). MR can accomplish this because genetic alleles are randomized at conception, analogous to how participants are assigned into groups in a randomized clinical trial.^9,10,11,12,13^ Indeed, the J-shaped association between BMI and all-cause mortality has been reproduced using MR, although it is not yet established how body composition and fat distribution contribute to the J-shaped curve.^9,10,12,14^

An optimal clinical adiposity measure would have a strong causal relationship with adverse outcomes, have consistency across the range of body composition, and be easy to measure. Additionally, it would lead to easy identification of adiposity targets causally associated with the lowest mortality risk. Hence, we perform a comparative analysis between 3 anthropometric adiposity measures (BMI, FMI, and WHR) with mortality from all-cause and specific causes, using observational and MR analyses.

## Methods

### Study Population

The UK Biobank (UKB) is a prospective cohort of more than 500 000 individuals between ages 40 and 69 years.^4^ The UKB data set issued on August 3, 2021, was used for analyses, including 408 160 unrelated White British individuals with genotypic data suitable for analyses. Of these, 20 065 participants with incomplete phenotypic data (eg, BMI, age, sex) and 423 participants with extreme BMI^3,9,15^ (<15 or >50) were excluded, arriving at a final sample size of 387 672. We partitioned the UKB population into 2 subsets: a discovery cohort (n = 337 078) and a validation cohort (n = 50 594) in preparation for deriving genetically determined adiposity measures through genome-wide association studies (GWAS) and polygenic risk score (PRS) computations. The validation cohort included all-cause mortality cases matched to living controls based on age, sex, and the first 10 principal components (genetic ancestry) according to propensity score, which equilibrates the covariate distribution between cases and controls (25 297 cases and 25 297 controls) while the discovery set included all remaining participants.^16^ The partition was done to achieve maximal power (eTable 2 in Supplement 1). Exposures included BMI, FMI, and WHR, derived from anthropometric weight and height, bioelectrical impedance analysis (BIA), and waist and hip circumference measurements respectively.^17^ FMI is the ratio between fat mass (measured in kilograms) and height (measured in meters).^2,17^ FMI was used instead of whole-body fat mass to adjust for height in an absolute indicator of fat mass–specific adiposity.^2^ There are no established FMI cutoff points for obesity.^17^ WHR is a unitless surrogate for abdominal adiposity, with cutoffs for obesity being greater than 0.85 for women and greater than 0.90 for men.^18^ The outcomes of interest were all-cause, cancer-related, cardiovascular disease (CVD)–related, and respiratory disease–related mortality, along with mortality resulting from all other diseases (other). Outcomes were defined based on the *International Statistical Classification of Diseases and Related Health Problems, Tenth Revision* (*ICD-10*) codes (eTable 4 in Supplement 1).^9^ Prevalent cases of CVD, cancer, and respiratory disease were excluded to avoid reverse causation in all analyses.^3^ Ethical approval for the UKB study was obtained from the North West Multi-center Research Ethics Committee. All participants provided informed consent. This study adhered to the Strengthening the Reporting of Observational Studies in Epidemiology (STROBE) reporting guidelines for MR studies.

### PRS Calculations

PRSs are quantitative measures of an individual’s genetic disposition to a certain trait, derived from the weighted effects of genetic variants (eg, single polynucleotide variants [SNVs]) on the phenotype.^19^ GWAS summary statistics for BMI and WHR were obtained from the Genetic Investigation of Anthropometric Traits (GIANT) while summary statistics for FMI were derived from the UKB.^4,19^ GIANT summary statistics were derived from the Locke et al^20^ meta-analysis, which excluded UKB participants.^4^ When deriving summary statistics within the UKB, the GWAS software REGENIE was used on our discovery cohort. REGENIE uses a linear mixed model to assess the association between genetic variants and a given trait, after adjusting for age, age squared, chip type, the first 40 genetic principal components, and UKB assessment center, as described elsewhere.^19,20,21,22^ To avoid overfitting, GWAS data for FMI were derived in the UKB discovery set only. PRS for each exposure were derived from their corresponding GWAS using LASSOSUM, a penalized regression method, including significantly associated genetic variants (P < .01).

### Statistical Analysis

#### Observational Analyses

Cox proportional hazard models were used to determine the association between adiposity measures (BMI, FMI, and WHR) and incident mortality outcomes. Analyses were adjusted for age, sex, and the first 10 genetic principal components. A fully adjusted model including smoking status, diabetes status, alcohol consumption, total cholesterol level, low-density lipoprotein cholesterol level, high-density lipoprotein cholesterol level, triglycerides level, systolic blood pressure, and diastolic blood pressure. An unadjusted model was also included. All covariates were chosen based on previous literature and association with adiposity (eTable 3 in Supplement 1).^3^ Sex- and age-stratified analyses with all-cause mortality were additionally conducted. Analysis of variance was used to determine whether a nonlinear model provided a better fit than a linear model (using an *F* statistic threshold of 10). All analyses were completed in the full UKB population (N = 387 672).

#### Linear MR

Linear MR was used to determine the strength of the association between adiposity measures (BMI, FMI, and WHR) vs incident mortality outcomes. Estimates were computed using an allelic scoring method and the Wald ratio. Analyses were performed using the same covariate schemes previously described from our observational analyses. As secondary analyses, models were stratified by sex, age, and presumed menopausal status. As described previously, female participants aged 52 years and younger were defined as premenopausal age, while postmenopausal age refers to female participants aged 53 years and older.^23^ MR-egger, inverse variance weighting (IVW), and weighted median were used for sensitivity analyses (eMethods in Supplement 1).^9,14^

#### Nonlinear MR

To assess the consistency requirement for the optimal adiposity measure, nonlinear MR (NLMR) was used to determine whether genetically determined adiposity-mortality associations varied across individual body composition.^9,15^ UKB participants were partitioned into 20 quantiles of measured BMI, FMI, or WHR after residualization for the corresponding PRS to avoid collider bias.^9^ Within each quantile, the Wald ratio for each respective PRS was computed. Ordinary least squares regression was used to determine whether there was variation in the Wald ratio between quantiles, testing for both linearity (standard linear regression) and nonlinearity (inclusion of a quadratic term). Consistency was defined as nonsignificant variation of the exposure-outcome association across quantiles. Analysis of variance was used to test statistically significant nonlinearity (*P* < .05). NLMR analyses were performed using the same covariate schemes previously described for linear MR.

All statistical analyses were conducted using R version 3.6.3 (R Project for Statistical Computing). Statistical significance was set at *P* < .05. Strength of association was quantified by test statistics such as β effect estimates, hazard ratios (HRs), and odds ratios (ORs). The generic inverse variance method was used for assessing heterogeneity between effect estimates from our linear epidemiological and MR analyses.

## Results

### Observational Associations Between Adiposity and Mortality

**Figure 1.**
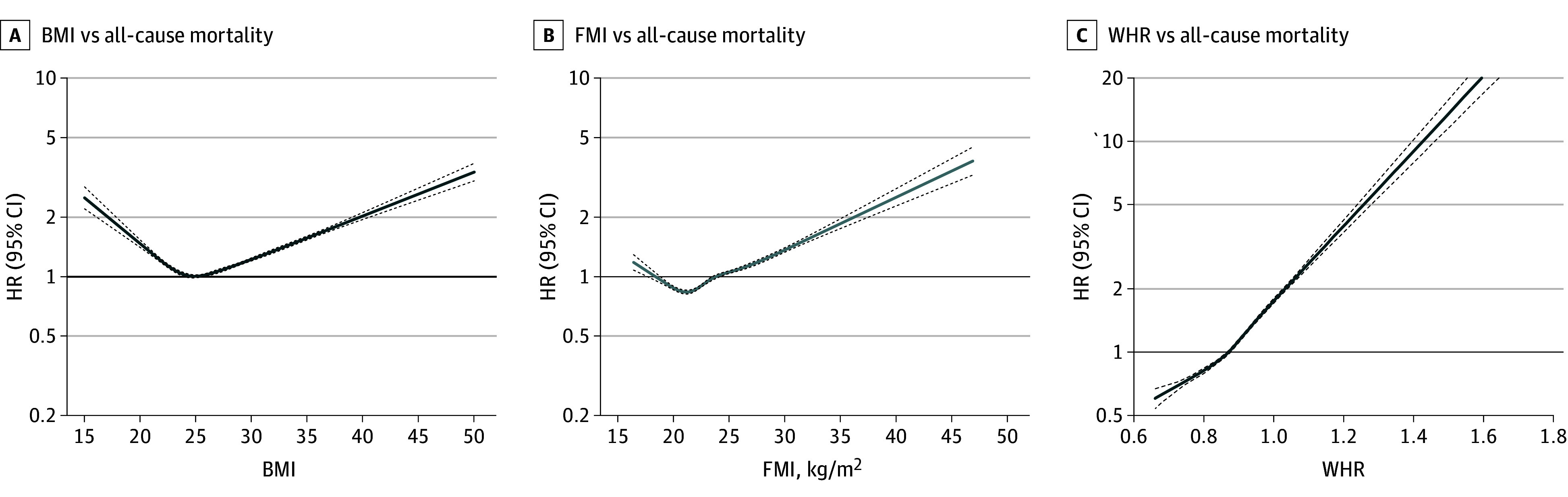
Association of Body Mass Index (BMI), Fat Mass Index (FMI), and Waist-to-Hip Ratio (WHR) With All-Cause Mortality Among 387 672 Participants from the UK Biobank BMI calculated as weight in kilograms divided by height in meters squared.

Overall, 387 672 and 50 594 participants were analyzed in our observational and MR analyses, respectively (observational analysis: mean [SD] age, 56.9 [8.0] years; 177 340 [45.7%] male; 210 332 [54.2%] female; MR analysis: mean [SD] age, 61.6 [6.2] years; 30 031 [59.3%] male; 20 563 [40.6%] female) (eTable 1 in Supplement 1). A J-shaped association was found of both measured BMI and FMI with all-cause mortality in the adjusted analysis, with the nadir at a BMI of 24.9 and an FMI of 6.15 (BMI, *P* for nonlinearity = 4.71 × 10^−169^; FMI, *P* for nonlinearity = 1.90 × 10^−33^) (Figure 1). A significant monotonically increasing association was found between WHR and all-cause mortality (Figure 1). Congruent results were obtained in the fully adjusted model, among male and female participants separately, and across age groups (eTable 13, eFigure 1, and eFigure 7 in Supplement 1).

Observational analyses showed that there were positive associations for all adiposity measures with cancer mortality (HR per SD change in BMI, 1.06 [95% CI, 1.04-1.08]; *P* = 3.52 × 10^−8^; HR per SD change in FMI, 1.08 [95% CI, 1.05-1.10]; *P* = 5.36 × 10^−11^; HR per SD change in WHR, 1.18 [95% CI, 1.15-1.21]; *P* = 1.47 × 10^−34^) and CVD mortality (HR per SD change in BMI, 1.41 [95% CI, 1.37-1.45]; *P* = 1.26 × 10^−131^; HR per SD change in FMI, 1.45 [95% CI, 1.40-1.49]; *P* = 4.61 × 10^−116^; HR per SD change in WHR, 1.59 [95% CI, 1.53-1.64]; *P* = 8.14 × 10^−155^). For BMI and FMI, there were J-shaped associations with mortality due to respiratory disease and other causes of mortality. For respiratory disease–associated mortality, the nadirs associated with the lowest rate of mortality were 26.0 and 7.43 for BMI and FMI, respectively. For the other mortality category, the nadirs for BMI and FMI were 25.5 and 6.55, respectively. In contrast, WHR exhibited monotonically increasing associations with cause-specific mortality (eFigure 2 in Supplement 1).

### MR Associations of Adiposity With Mortality

**Figure 2.**
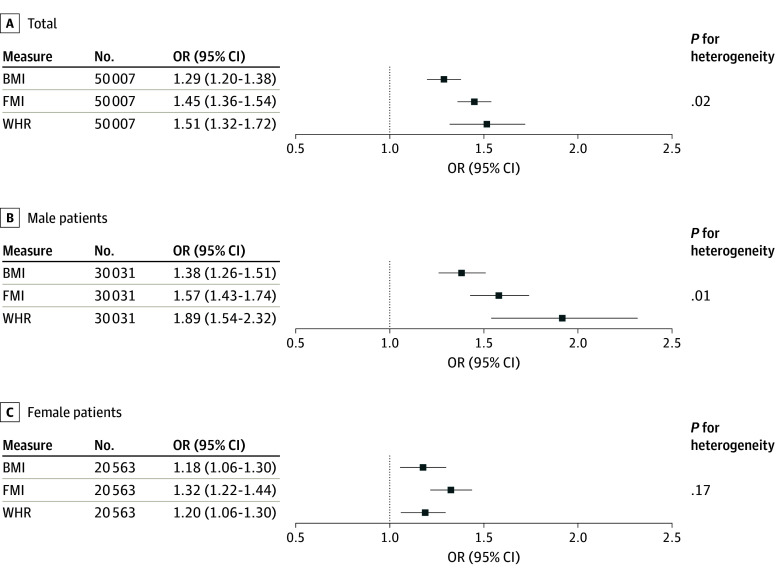
Linear Mendelian Randomization Analyses Comparing Genetically Determined Adiposity Measures in Their Association With All-Cause Mortality BMI indicates body mass index; FMI, fat mass index; OR, odds ratio; and WHR, waist-to-hip ratio.

To verify the relevance of PRS, we confirmed that all PRSs were significantly associated with their corresponding traits (eTable 5 in Supplement 1). Our observational analyses showed that BMI and FMI had J-shaped associations with all-cause mortality, while WHR had a linear association. Thus, the only way we could compare the strength of association between adiposity measures was through their linear estimates. There was a significant association between all 3 adiposity measures and all-cause mortality (OR per SD change in genetically determined BMI, 1.29 [95% CI, 1.20-1.38]; *P* = 1.44 × 10^−13^; OR per SD change in genetically determined FMI, 1.45 [95% CI, 1.36-1.54]; *P* = 6.27 × 10^−30^; OR per SD change in genetically determined WHR, 1.51 [95% CI, 1.32-1.72]; *P* = 2.11 × 10^−9^) (Table 1). Compared with BMI, WHR had the stronger association with all-cause mortality, although it was not significantly stronger than FMI (*I*^2^ = 74.8%; *P* = .02 for all comparisons except between FMI and WHR) (Figure 2). Associations were significant irrespective of adjustment (Table 1). Compared with epidemiological analyses, estimates for the adiposity-mortality associations were stronger using MR in the adjusted models (eFigures 3-5 in Supplement 1).

**Table 1.** Summary of Epidemiological and Linear MR Analyses of Adiposity Measures and All-Cause Mortality

All-cause mortality^a^	Effect size per SD change in BMI (95% CI)^b^	Effect size per SD change in FMI (95% CI)^b^	Effect size per SD change in WHR (95% CI)^b^
**Adjusted**			
MR, OR	1.29 (1.20-1.38)	1.45 (1.36-1.54)	1.51 (1.32-1.72)
Epidemiological, HR	1.14 (1.13-1.16)	1.15 (1.13-1.17)	1.41 (1.38-1.43)
**Unadjusted**			
MR, OR	1.29 (1.11-1.50)	1.47 (1.33-1.62)	1.40 (1.19-1.65)
Epidemiological, HR	1.17 (1.15-1.18)	1.06 (1.04-1.08)	1.52 (1.51-1.54)
**Males**			
MR, OR	1.38 (1.26-1.51)	1.57 (1.43-1.74)	1.89 (1.54-2.32)
Epidemiological, HR	1.16 (1.14-1.18)	1.23 (1.21-1.26)	1.43 (1.41-1.46)
**Females**			
MR, OR	1.18 (1.06-1.30)	1.32 (1.22-1.44)	1.20 (1.06-1.30)
Epidemiological, HR	1.13 (1.11-1.15)	1.14 (1.12-1.16)	1.37 (1.34-1.40)

Abbreviations: MR, mendelian randomization; BMI, body mass index; FMI, fat mass index; HR, hazard ratio; OR, odds ratio; WHR, waist-to-hip ratio.

^a^ MR analyses had 50 594 participants in the adjusted model with male and female participants combined; 30 031, male-only cohort; and 20 563, female-only cohort. Epidemiological analyses had 387 672 participants in the adjusted model with male and female participants combined; 177 324, male-only cohort; 210 319, female-only cohort.

^b^ Effect sizes are ORs for MR analyses and HRs for epidemiological analyses. HRs indicate the association of a 1-SD unit increase in adiposity measure with risk of all-cause mortality. ORs indicate the association of a 1-SD unit increase in genetically determined adiposity measure with risk of all-cause mortality. The adjusted model was used for all sex-specific analyses. All associations had *P* < .05.

Given that there was significant interaction between sex and genetically determined adiposity measures with mortality outcomes (Table 1), we computed sex-stratified analyses. A significant association between WHR and all-cause mortality was observed in male participants (OR per SD change, 1.89 [95% CI, 1.54-2.32]; *P* = 1.44 × 10^−9^) and female participants (OR per SD change, 1.20 [95% CI, 1.01-1.43]; *P* = 3.77 × 10^−2^; *P* for interaction = .02). In male participants, WHR had a stronger association with all-cause mortality than BMI (*I*^2^ = 86.7%; *P* = .006) (Table 1 and Figure 2). In female participants, all adiposity measures were similarly associated with all-cause mortality (*I*^2^ = 43.6%; *P* = .17) (Table 1 and Figure 2). There was no significant difference between female participants of premenopausal or postmenopausal age in the association of adiposity measures with all-cause mortality (*I*^2^ = 0%; *P* = .77; *I*^2^ = 37.1%; *P* = .20) (eTable 6 in Supplement 1).

Our epidemiological and MR analyses found that there was no significant difference between age groups in the BMI–all-cause mortality association (epidemiological: *I*^2^ = 0%; *P* = .90; MR: *I*^2^ = 58.9%; *P* = .05). Among MR analyses, there was attenuation of the FMI–all-cause mortality association when comparing estimates in the group aged 49 to 56 years with that older than 64 years group (age 49-56 years: OR, 1.89 [95% CI, 1.58-2.26]; age >64 years: OR, 1.16 [95% CI, 1.14-1.19], *I*^2^ = 95.1%; *P* = 5.87 × 10^−6^). Epidemiological and MR analyses found that the WHR–all-cause mortality association also attenuated with age (age 49-56 years, epidemiological: OR, 1.43 [95% CI, 1.39-1.48]; age >64 years, epidemiological: OR, 1.37 [95% CI, 1.34-1.40]; *I*^2^ = 99.1%; *P* = 6.04 × 10^−98^; age 49-56 years, MR: OR, 2.44 [95% CI, 1.68-3.53]; age >64 years, MR: OR, 1.53 [95% CI, 1.24-1.89]; *I*^2^ = 95.1%; *P* < .001) (eFigures 8-10 and eTable 14 in Supplement 1).

Regarding our MR analyses with cause-specific mortality outcomes, all adiposity measures were significantly associated with mortality due to CVD or other diseases (CVD: OR, 1.30 [95% CI, 1.15-1.47]; *P* < .001; other diseases: OR, 1.26 [95% CI, 1.17-1.34]; *P* < .001 (Table 2). Only FMI was significantly associated with respiratory disease mortality (OR, 1.33 [95% CI, 1.12-1.59; *P* < .001) (Table 2). Apart from the FMI–cancer mortality association (MR-egger estimated OR, 0.99; 95% CI, 0.92-1.07; *P* = .03), most results did not show evidence of pleiotropy using Egger, inverse weighted variance, and weighted median sensitivity analyses (eTables 7-12 in Supplement 1).

**Table 2.** Summary of Epidemiological and Linear MR Analyses of Adiposity Measures and Cause-Specific Mortality

Cause-specific mortality^a^	Effect size per SD change in BMI (95% CI)^b^	*P* value	Effect size per SD change in FMI (95% CI)^b^	*P* value	Effect size per SD change in WHR (95% CI)^b^	*P* value
Cancer						
MR, OR	1.05 (0.97-1.15)	.23	1.01 (0.93-1.09)	.84	1.04 (0.87-1.23)	.68
Epidemiological, HR	1.06 (1.04-1.08)	<.001	1.07 (1.05-1.09)	<.001	1.18 (1.15-1.21)	<.001
CVD						
MR, OR	1.30 (1.15-1.47)	<.001	1.38 (1.23-1.55)	<.001	1.56 (1.23-1.99)	<.001
Epidemiological, HR	1.41 (1.37-1.45)	<.001	1.45 (1.39-1.51)	<.001	1.59 (1.53-1.64)	<.001
Respiratory						
MR, OR	1.00 (0.83-1.19)	.96	1.33 (1.12-1.59)	<.001	1.11 (0.77-1.60)	.57
Epidemiological, HR	0.91 (0.87-0.96)	<.001	0.99 (0.93-1.05)	<.001	1.26 (1.18-1.34)	<.001
Other						
MR, OR	1.26 (1.17-1.34)	<.001	1.40 (1.31-1.49)	<.001	1.39 (1.22-1.60)	<.001
Epidemiological, HR	1.10 (1.07-1.14)	<.001	1.12 (1.07-1.16)	<.001	1.32 (1.27-1.37)	<.001

Abbreviations: BMI, body mass index; CVD, cardiovascular disease; FMI, fat mass index; HR, hazard ratio; MR, mendelian randomization; OR, odds ratio; WHR, waist-to-hip ratio.

^a^ MR analyses included 50 594 participants; epidemiological analyses, 387 672.

^b^ Effect sizes are OR for MR analyses and HR for epidemiological analyses. HRs indicate the association of a 1-SD unit increase in adiposity measure with risk of cause-specific mortality. ORs indicate the association of a 1-SD unit increase in genetically determined adiposity measure with risk of cause-specific mortality. The adjusted model was used for all analyses involving cause-specific mortality.

### Nonlinear Mendelian Randomization Analyses

The association of genetically determined BMI and FMI with all-cause mortality varied across quantiles of observed BMI, but WHR did not (BMI: β = −0.003; *P* = .04; FMI: β = 0.03; *P* = .02; and WHR: β = −0.001; *P* = .58). Data appear in eFigure 6 in Supplement 1.

## Discussion

An optimal marker of adiposity is easy to measure and meets the following characteristics: causal relationships, strong association with mortality, and consistency across adiposity levels, as measured by BMI. Our study found that the association between WHR and all-cause mortality was potentially causal, stronger than BMI, and consistent across quantiles of BMI.^24^ WHR is easier to measure than FMI due to FMI requiring BIA, which can be expensive and inaccessible.^17,24^

Our MR findings support a possible causal relationship between WHR and mortality. This suggests that not only can WHR be used as clinical risk marker, but it may be a suitable intervention target, given weight loss is associated with a lower WHR.^25^ This is congruent with previous literature showing WHR was strongly associated with disease outcomes linked with top causes of death.^7^ We also found an association using MR between WHR and mortality from both CVD and other diseases, broadly congruent with another study that found genetically determined WHR was associated with higher levels of triglycerides, type 2 diabetes, and coronary heart disease risk.^10^ These findings provide a potential causal pathway linking WHR to mortality, especially CVD mortality.^9,26^ Nevertheless, having a causal relationship with mortality is not sufficient for an adiposity measure to be considered optimal. Indeed, causal relationships for BMI and FMI with all-cause mortality were also supported by MR.

Both our epidemiological and MR analyses also demonstrated that WHR had a stronger association with all-cause mortality than FMI or BMI. This result is congruent with previous literature where BMI, although widely used in clinical practice, has been shown to have a weaker association with disease or mortality risk compared with FMI or WHR.^9,10,11,12^ In one MR study examining the causal effect of genetically determined BMI and WHR on blood pressure traits,^27^ WHR was found to have a larger effect than BMI, despite both measures being significantly associated with these traits.

An optimal marker of adiposity should also provide consistent associations across different adiposity levels, as measured by BMI. Contrary to BMI and FMI, the WHR-mortality association did not differ across BMI quantiles. When contextualizing this result to the lower end of the J-shaped BMI-mortality curve, our results show that increased abdominal fat mass is still detrimental to health, despite low BMI. Previous studies have postulated that the lower end of the J-shape curve could be driven by lean mass loss due to disease-related cachexia or malnutrition, regardless of the fat mass and/or abdominal adiposity quantity still present.^7^ Nevertheless, it is because of the curvilinear shape that both BMI and FMI are less ideal measures of adiposity, independent of their strength of association with mortality. Use of WHR alongside BMI may help improve risk stratification for patients, with further evidence for incorporation of WHR as a primary outcome for future clinical interventions.^24^

Adiposity traits are known to be sexually dimorphic.^7,28,29^ Females have higher SNV-based heritability for WHR and larger effect sizes in more than 90% of WHR (adjusted for BMI)–associated SNVs compared with males.^7^ Contrary to previous literature, our study showed that males with a higher WHR had a disproportionately higher risk of mortality than females. Thus, assessing and targeting WHR may have greater relevance in males. Since female adiposity distribution is drastically different than that in males, the relatively higher abdominal obesity in males may explain the sex-specific difference in risk.^2,7,24^ A previous MR study showed that increased WHR was linked with larger effects on risk of kidney outcomes and chronic obstructive pulmonary disease, leading causes of death, in males compared with females.^7^ We could not assess whether these diseases were driving the sex-specific differences due to limited power. No difference was observed between female participants of premenopausal and postmenopausal age, although we were limited by a relatively small sample size in the premenopausal aged group.

A previous study found that the BMI–all-cause mortality association weakened with older age.^3^ While our MR results contrast from the literature in that the BMI–all-cause mortality association did not attenuate with age, the FMI– and WHR–all-cause mortality associations did attenuate with age. Bhaskaran et al^3^ mentioned that the reason why the BMI–all-cause mortality association would attenuate is because nutritional reserves become more important with age. They also mentioned their results may have been due to reverse causality, as there is increased prevalence of major disease among older individuals, some impacting BMI due to muscle mass loss.^3^ As WHR is a proxy of central adiposity, this may be the component of FMI and BMI that is driving the attenuation of the BMI–all-cause mortality association. However, future studies are needed to investigate the biological correlate of these observations.

### Limitations

This study has limitations. We used a genetically homogeneous and unrelated White British population; thus, our results may not be representative of other populations. Future studies in larger non-European populations should be conducted to see whether our findings persist in other racial and ethnic groups.^6^ All adiposity measures were assessed at baseline, and we could not assess whether changes in adiposity over time impact mortality. While the UKB does have follow-up data on some adiposity measures, the sample size is relatively small and not amenable to such an investigation. Our optimal adiposity marker definition might not be complete: we may have missed elements that would lead to the most accurate definition. Additionally, as BMI and FMI have nonlinear associations with mortality, our comparisons using their linear effect estimates may not be representative of their true effect on mortality.

## Conclusions

In this cohort study, compared with BMI, WHR had the strongest, most robust, and consistent association with all-cause mortality and was the only measurement unaffected by BMI. Current WHO recommendations for optimal BMI range are inaccurate across individuals with various body compositions and therefore suboptimal for clinical guidelines. Future research is needed to explore whether using WHR as the primary clinical measure of adiposity would help to improve long-term health outcomes in distinct patient populations compared with BMI. Our results provide further support to shift public health focus from measures of general adiposity, such as BMI, to adiposity distribution using WHR.

## Notes

Supplement 1. **eTable 1.** Baseline Characteristics of Participants in the UKB **eTable 2.** List of GWAS Consortia **eMethods.** UKB Population and Extended Methods **eTable 3.** BMI Association With Covariates **eTable 4.** Phenotype and Mortality Outcome Definitions **eTable 5.** Validation of BMI, FMI, and WHR PRS **eFigure 1.** Association of BMI, FMI, and WHR With All-Cause Mortality in All UKB Participants (N = 387 672), Males (n = 177 340), and Females (n = 210 332) **eFigure 2.** Association of BMI, FMI, and WHR With Cause-Specific Mortality Outcomes **eTable 6.** Linear Mendelian Randomization Analyses Comparing the Association of Individual Genetically Determined Adiposity Measures With All-Cause Mortality Between Premenopausal vs Postmenopausal Females in the UKB **eFigure 3.** Comparison Between Epidemiologically Derived and MR Derived Estimates for the BMI–All-Cause Mortality Association **eFigure 4.** Comparison Between Epidemiologically Derived and MR-Derived Estimates for the FMI–All-Cause Mortality Association **eFigure 5.** Comparison Between Epidemiologically Derived and MR-Derived Estimates for the WHR–All-Cause Mortality Association **eTable 7.** Select Single Nucleotide Variants (SNVs) Associated With BMI **eTable 8.** Select Single Nucleotide Variants (SNVs) Associated With FMI **eTable 9.** Select Single Nucleotide Variants (SNVs) Associated With WHR **eTable 10.** Egger Regression Mendelian Randomization Analyses **eTable 11.** Inverse Variance Weighted Mendelian Randomization Analyses **eTable 12.** Weighted Median Mendelian Randomization Analyses **eTable 13.** Summary of Epidemiological and Linear Mendelian Randomization Analyses of Adiposity Measures and All-Cause Mortality: Full Adjusted Model **eFigure 6.** Nonlinear Mendelian Randomization Analyses Comparing Genetically Determined Adiposity Measure–All-Cause Mortality Associations Across BMI Quantiles **eFigure 7.** Epidemiological Association Between BMI, FMI, and WHR and All-Cause Mortality Across Age Groups **eTable 14.** Summary of Epidemiological and Linear Mendelian Randomization Analyses of Adiposity Measures and All-Cause Mortality Across Age Groups **eFigure 8.** Comparison Between Epidemiologically Derived and MR-Derived Estimates for the BMI–All-Cause Mortality Association Between Age Groups **eFigure 9.** Comparison Between Epidemiologically Derived and MR-Derived Estimates for the FMI–All-Cause Mortality Association Between Age Groups **eFigure 10.** Comparison Between Epidemiologically Derived and MR-Derived Estimates for the WHR–All-Cause Mortality Association Between Age Groups **eTable 15.** Comparison Between the Cox Regression and Logistic Regression Model in the Adjusted Linear Mendelian Randomization Analysis **eTable 16.** Comparison Between the Cox Regression and Logistic Regression Model in the Adjusted Observational Analysis
eReferences.

Supplement 2.
Data Sharing Statement
