# Error in Methods

**DOI:** 10.1001/jamanetworkopen.2025.0472

**Published:** 2025-02-04

In the Original Investigation titled “Surrogate Adiposity Markers and Mortality,”^1^ published September 20, 2023, there was an error in the Methods section. In the “Study Population” subsection, the waist-to-hip ratio cutoff for obesity for men should have read 0.90. This article has been corrected.^1^

## References

[1] Khan I, Chong M, Le A, . Surrogate adiposity markers and mortality. JAMA Netw Open. 2023;6(9):e2334836. doi:10.1001/jamanetworkopen.2023.34836 37728925 PMC10512100

