# Supplementary material for: Surrogate Adiposity Markers and Mortality
Source: JAMA Netw Open. 2023 Sep 20;6(9):e2334836. doi: 10.1001/jamanetworkopen.2023.34836 (PMC10512100; doi:10.1001/jamanetworkopen.2023.34836)
Supplement: Supplement 1. — eTable 1. Baseline Characteristics of Participants in the UKB eTable 2. List of GWAS Consortia eMethods. UKB Population and Extended Methods eTable 3. BMI Association With Covariates eTable 4. Phenotype and Mortality Outcome Definitions eTable 5. Validation of BMI, FMI, and WHR PRS eFigure 1. Association of BMI, FMI, and WHR With All-Cause Mortality in All UKB Participants (N = 387 672), Males (n = 177 340), and Females (n = 210 332) eFigure 2. Association of BMI, FMI, and WHR With Cause-Specific Mortality Outcomes eTable 6. Linear Mendelian Randomization Analyses Comparing the Association of Individual Genetically Determined Adiposity Measures With All-Cause Mortality Between Premenopausal vs Postmenopausal Females in the UKB eFigure 3. Comparison Between Epidemiologically Derived and MR Derived Estimates for the BMI–All-Cause Mortality Association eFigure 4. Comparison Between Epidemiologically Derived and MR-Derived Estimates for the FMI–All-Cause Mortality Association eFigure 5. Comparison Between Epidemiologically Derived and MR-Derived Estimates for the WHR–All-Cause Mortality Association eTable 7. Select Single Nucleotide Variants (SNVs) Associated With BMI eTable 8. Select Single Nucleotide Variants (SNVs) Associated With FMI eTable 9. Select Single Nucleotide Variants (SNVs) Associated With WHR eTable 10. Egger Regression Mendelian Randomization Analyses eTable 11. Inverse Variance Weighted Mendelian Randomization Analyses eTable 12. Weighted Median Mendelian Randomization Analyses eTable 13. Summary of Epidemiological and Linear Mendelian Randomization Analyses of Adiposity Measures and All-Cause Mortality: Full Adjusted Model eFigure 6. Nonlinear Mendelian Randomization Analyses Comparing Genetically Determined Adiposity Measure–All-Cause Mortality Associations Across BMI Quantiles eFigure 7. Epidemiological Association Between BMI, FMI, and WHR and All-Cause Mortality Across Age Groups eTable 14. Summary of Epidemiological and Linear Mendelian Randomizat [file jamanetwopen-e2334836-s001.pdf]

Supplemental Online Content

Khan I, Chong M, Le A, et al. Surrogate adiposity markers and mortality among White adults. *JAMA Netw Open.* 2023;6(9):e2334836. doi:10.1001/jamanetworkopen.2023.34836

eTable 1. Baseline Characteristics of Participants in the UKB

eTable 2. List of GWAS Consortia

eMethods. UKB Population and Extended Methods

eTable 3. BMI Association With Covariates

eTable 4. Phenotype and Mortality Outcome Definitions

eTable 5. Association of BMI, FMI, and WHR With BMI, FMI, and WHR PRS

eFigure 1. Association of BMI, FMI, and WHR With All-Cause Mortality in All UKB Participants (N = 387 672), Males (n = 177 340), and Females (n = 210 332)

eFigure 2. Association of BMI, FMI, and WHR With Cause-Specific Mortality Outcomes

eTable 6. Linear Mendelian Randomization Analyses Comparing the Association of Individual Genetically Determined Adiposity Measures With All-Cause Mortality Between Premenopausal vs Postmenopausal Females in the UKB

eFigure 3. Comparison Between Epidemiologically Derived and MR Derived Estimates for the BMI–All-Cause Mortality Association

eFigure 4. Comparison Between Epidemiologically Derived and MR-Derived Estimates for the FMI–All-Cause Mortality Association

eFigure 5. Comparison Between Epidemiologically Derived and MR-Derived Estimates for the WHR–All-Cause Mortality Association

eTable 7. Select Single Nucleotide Variants (SNVs) Associated With BMI

eTable 8. Select Single Nucleotide Variants (SNVs) Associated With FMI

eTable 9. Select Single Nucleotide Variants (SNVs) Associated With WHR

eTable 10. Egger Regression Mendelian Randomization Analyses

eTable 11. Inverse Variance Weighted Mendelian Randomization Analyses

eTable 12. Weighted Median Mendelian Randomization Analyses

eTable 13. Summary of Epidemiological and Linear Mendelian Randomization Analyses of Adiposity Measures and All-Cause Mortality: Full Adjusted Model

eFigure 6. Nonlinear Mendelian Randomization Analyses Comparing Genetically Determined Adiposity Measure–All-Cause Mortality Associations Across BMI Quantiles

eFigure 7. Epidemiological Association Between BMI, FMI, and WHR and All-Cause Mortality Across Age Groups

© 2023 Khan I et al. *JAMA Network Open.*

eTable 14. Summary of Epidemiological and Linear Mendelian Randomization Analyses of Adiposity Measures and All-Cause Mortality Across Age Groups

eFigure 8. Comparison Between Epidemiologically Derived and MR-Derived Estimates for the BMI–All-Cause Mortality Association Between Age Groups

eFigure 9. Comparison Between Epidemiologically Derived and MR-Derived Estimates for the FMI–All-Cause Mortality Association Between Age Groups

eFigure 10. Comparison Between Epidemiologically Derived and MR-Derived Estimates for the WHR–All-Cause Mortality Association Between Age Groups

eTable 15. Comparison Between the Cox Regression and Logistic Regression Model in the Adjusted Linear Mendelian Randomization Analysis

eTable 16. Comparison Between the Cox Regression and Logistic Regression Model in the Adjusted Observational Analysis

eReferences.

This supplemental material has been provided by the authors to give readers additional information about their work.

© 2023 Khan I et al. *JAMA Network Open.*

eTable 1. Baseline Characteristics of Participants in the UKB

Whole UKB Population

Baseline characteristics	All UKB participants	All deaths	Controls[a]
Number of participants	387,672	25,297	362,375
Total number of men	177,340	15,016	162,324
Total number of women	210,332	10,281	200,051
Mean (SD) age at baseline (years)	56.9 (8.0)	61.8 (6.3)	56.6 (8.0)
Mean (SD) body mass index (kg/m^2)	27.4 (4.6)	28.1 (5.1)	27.3 (4.6)
Mean (SD) whole body fat mass (kg)	24.8 (9.3)	25.6 (10.0)	24.8 (9.3)
Mean (SD) height (m)	1.69 (9.23)	1.69 (9.22)	1.69 (9.23)
Mean (SD) fat mass index (kg/m^2)	8.83 (3.55)	9.06 (3.74)	8.82 (3.55)
Mean (SD) waist-to-hip ratio	0.87 (0.1)	0.91 (0.1)	0.87 (0.1)
Death due to cancer	N/A	9,732	377,940
Death due to cardiovascular disease (CVD)	N/A	4,231	383,441
Death due to respiratory disease	N/A	1,755	385,917
Death due to non-cancer, CVD, or respiratory disease (“other”)	N/A	4,653	383,019

[a]The pure control group, which excludes all mortality cases and controls matched to mortality cases.

Case-Control Sample (i.e. The Testing or Validation Set)

Baseline characteristics	All UKB participants	All deaths	Controls[a]
Number of participants	50,594	25,297	25,297
Percentage (%) of men	30,031 (59.3)	15,016 (59.3)	15,015 (59.4)
Mean (SD) age at baseline (years)	61.6 (6.2)	61.8 (6.2)	61.3 (6.1)

© 2023 Khan I et al. *JAMA Network Open.*

Mean (SD) body mass index (kg/m^2)	27.9 (4.8)	28.2 (5.1)	27.6 (4.4)
Mean (SD) whole body fat mass (kg)	25.1 (9.5)	25.7 (10.0)	24.5 (8.9)
Mean (SD) height (m)	1.70 (9.19)	1.69 (9.22)	1.70 (9.16)
Mean (SD) fat mass index (kg/m^2)	8.83 (3.57)	9.06 (3.74)	8.61 (3.37)
Mean (SD) waist-to-hip ratio	0.90 (0.1)	0.91 (0.1)	0.89 (0.1)
Death due to cancer	N/A	9,732	40,862
Death due to cardiovascular disease (CVD)	N/A	4,231	46,363
Death due to respiratory disease	N/A	1,755	48,839
Death due to non-cancer, CVD, or respiratory disease ("other")	N/A	4,653	45,941

© 2023 Khan I et al. *JAMA Network Open.*

eTable 2. List of GWAS Consortia

Phenotype	Consortia Name
BMI	Genetic Investigation of ANthropometric Traits (GIANT)[1]
Waist-to-hip ratio (WHR)	Genetic Investigation of ANthropometric Traits (GIANT)[1]

© 2023 Khan I et al. *JAMA Network Open.*

eMethods. UKB Population and Extended Methods

UKB Population

We used the UKB dataset issued on August 3rd, 2021 as part of approved application #15255. Samples were excluded based on criteria from the standard UKB genomic analysis exclusion list (i.e. UKB data showcase data-field #22010)[13]. Further quality control (QC) was also done: related samples, samples with discordant reported sex versus genetic sex, lacking British ancestry, and with QC failure in the UK BiLEVE array were removed[13]. SNPs that had low minor allele frequency (MAF < 0.01), low quality of imputation (INFOscore < 0.6), deviation from Hardy-Weinberg equilibrium/poor genotype calling (HWE; $P < 10^{-6}$) or low call rates (< 99%) were removed[13].

Schematic representation of sample eligibility criteria and partitioning:

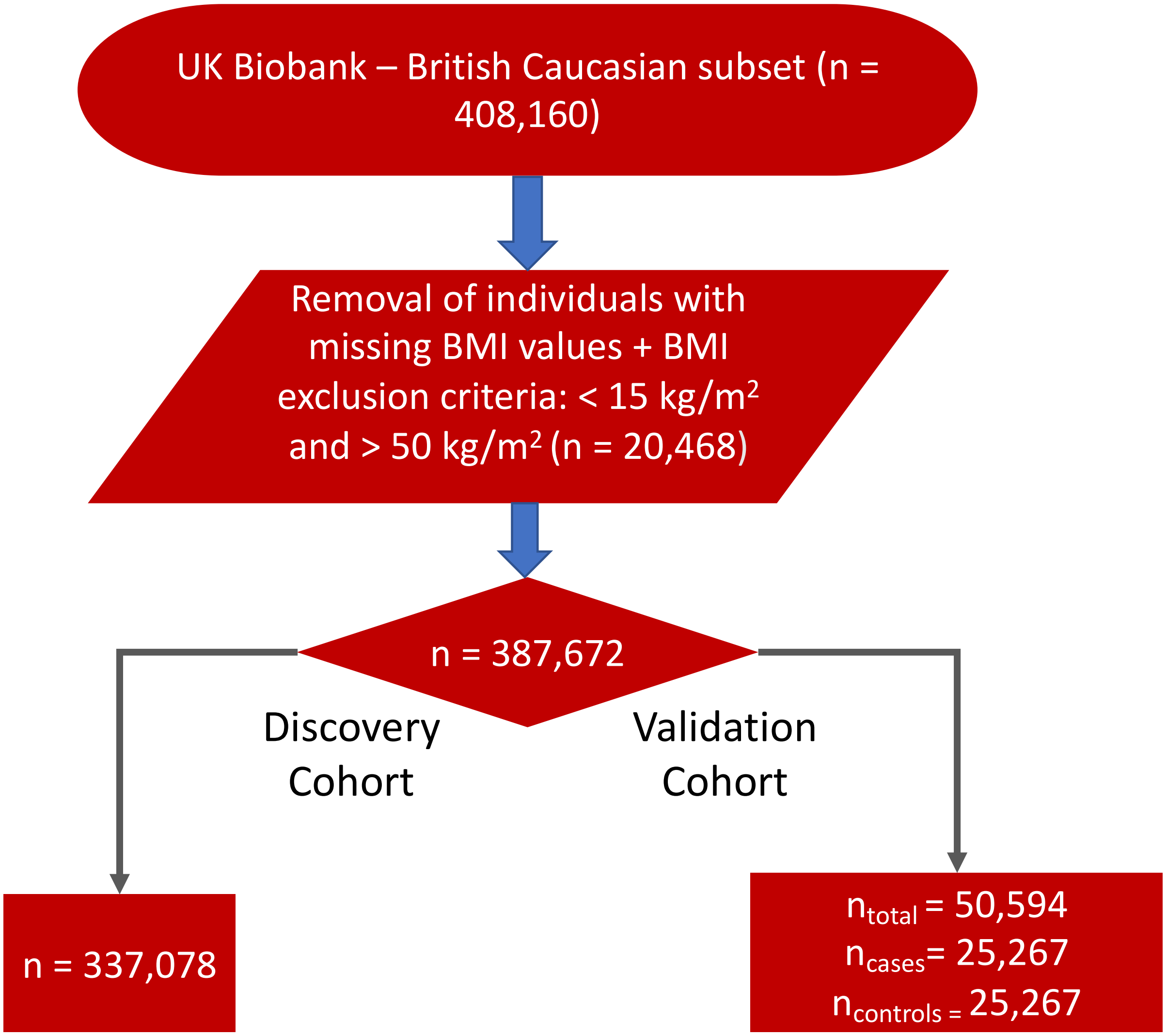

UK Biobank-Based Genome-Wide Association Studies

© 2023 Khan I et al. *JAMA Network Open.*

REGENIE, a program that uses whole genome regression modelling to run GWAS analyses, was used to compute GWAS within a select subset of UKB participants for fat mass index (FMI or whole body fat mass/height^2). REGENIE uses linear regression to assess the association between genetic variants and a given trait, after adjusting for age, age^2, chip type, the first 40 genetic principal components, and UKB assessment centre, as described elsewhere[9,19].

Polygenic risk scores

PRS	Number of SNPs included (at $p < 0.01$)
BMI	834,633
FMI	772,784
WHR	602,314

Linkage disequilibrium pruning for mendelian randomization analyses

PLINK was used to prune SNPs in linkage disequilibrium (LD) within a particular window. Our reference panel was the European 1000G panel[17].

eTable 3. BMI Association With Covariates[a]

Covariate ~ BMI Association	β/OR	*P* value	Adjusted R-squared
Age ~ BMI	0.01	0.09	3.67×10^{-5}
Sex ~ BMI	0.02	1.03×10^{-37} [b]	N/A
PC1 ~ BMI	-0.003	0.02 [b]	8.11×10^{-5}
PC2 ~ BMI	-0.0002	0.86	-1.91×10^{-5}
PC3 ~ BMI	-0.001	0.32	-5.74×10^{-7}
PC4 ~ BMI	0.01	0.0005 [b]	0.0002
PC5 ~ BMI	0.03	5.42×10^{-5} [b]	0.0003
PC6 ~ BMI	-0.003	0.05	5.82×10^{-5}
PC7 ~ BMI	0.0006	0.69	-1.67×10^{-5}
PC8 ~ BMI	-0.002	0.20	1.24×10^{-5}
PC9 ~ BMI	-0.01	0.02 [b]	9.43×10^{-5}
PC10 ~ BMI	0.002	0.44	-8.05×10^{-6}

[a] Estimates derived from the testing/validation cohort.
[b] Significance set at $p < 0.05$.

© 2023 Khan I et al. *JAMA Network Open.*

eTable 4. Phenotype and Mortality Outcome Definitions

Phenotype	UKB Field ID/ICD-10 Code
BMI	21001-0.0
Sex	31-0.0
Age	21022-0.0
FMI	23100-0.0 and 50-0.0
WHR	48-0.0 and 49-0.0
Height	50-0.0

Mortality Outcome	UKB Field ID/ICD-10 Code
All-Cause Mortality	40000-0.0
Cardiovascular Mortality	ICD-10 Code: I
Cancer Mortality	ICD-10 Code: C
Respiratory Disease Mortality	ICD-10 Codes: J00-09, J10-19, J20-22, J23-29, J3-9

© 2023 Khan I et al. *JAMA Network Open.*

eTable 5. Association of BMI, FMI, and WHR With BMI, FMI, and WHR PRS[a].

All regression analyses were adjusted for PRS other than the one being analyzed (e.g. the BMI ~ BMI PRS analysis was adjusted for FMI PRS and WHR PRS), age, sex, and the first 10 principal components.

**Phenotype ~ BMI PRS Association**	**β**	***P* value**
BMI ~ BMI PRS	0.20	0.004 [b]
FMI ~ BMI PRS	0.09	0.005 [b]
WHR ~ BMI PRS	-0.04	

**Phenotype ~ FMI PRS Association**	**β**	***P* value**
BMI ~ FMI PRS	0.20	0.005 [b]
FMI ~ FMI PRS	0.25	
WHR ~ FMI PRS	0.08	

**Phenotype ~ WHR PRS Association**	**β**	***P* value**
BMI ~ WHR PRS	0.02	0.007 [b]
FMI ~ WHR PRS	0.03	
WHR ~ WHR PRS	0.25	

[a]Estimates derived from the testing/validation cohort.
[b] Significance set at $p < 0.05$.

© 2023 Khan I et al. *JAMA Network Open.*

eFigure 1. Association of BMI, FMI, and WHR With All-Cause Mortality in All UKB Participants (N = 387 672), Males (n = 177 340), and Females (n = 210 332)

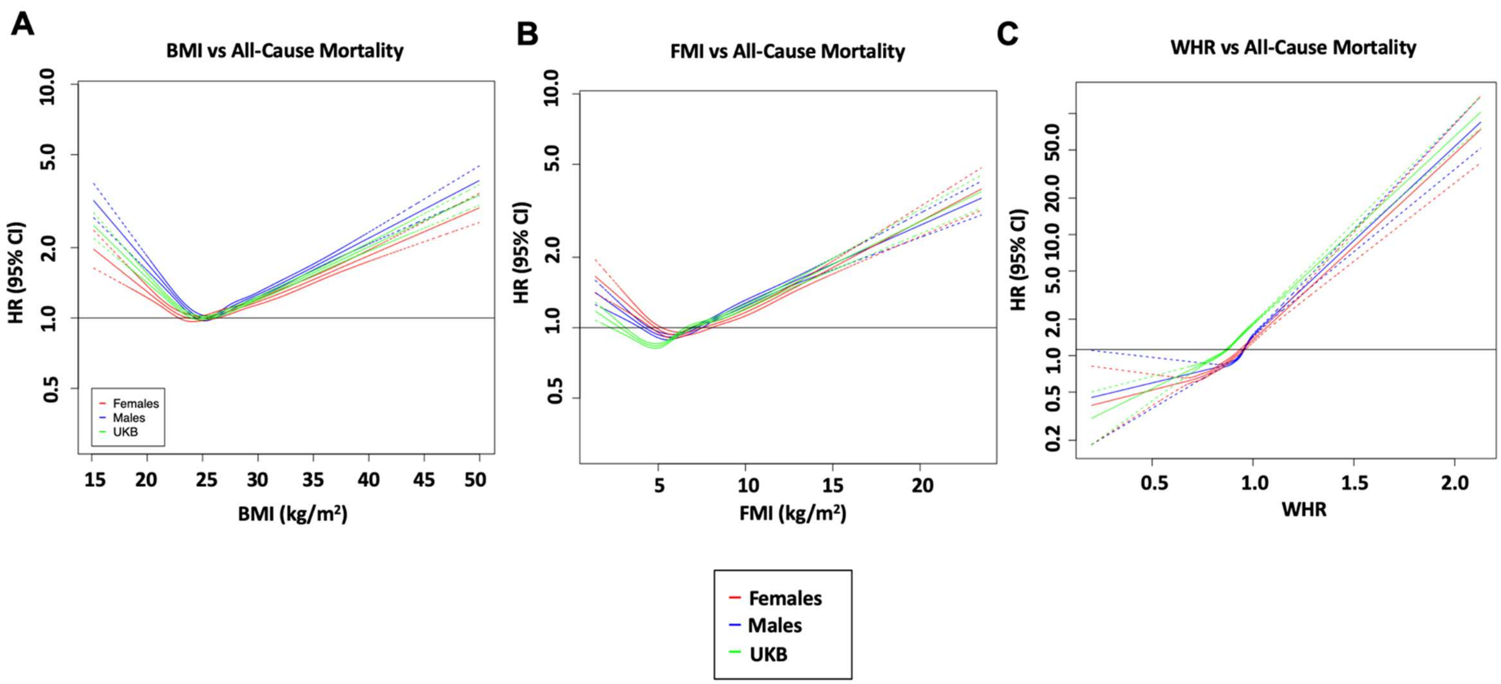

BMI = body mass index, FMI = fat mass index, WHR = Waist-to-hip ratio, HR = hazard ratio for all-cause mortality. Statistical significance for non-linearity Bonferroni-corrected to $p < 0.05$. The reference point at HR = 1 for BMI (25 kg/m^2), the mean value for FMI in the UKB population (8.83 kg/m^2), and the mean value for WHR in the UKB population (0.87) for analyses with BMI, FMI, and WHR were used as independent variables, respectively.

© 2023 Khan I et al. *JAMA Network Open.*

eFigure 2. Association of BMI, FMI, and WHR With Cause-Specific Mortality Outcomes

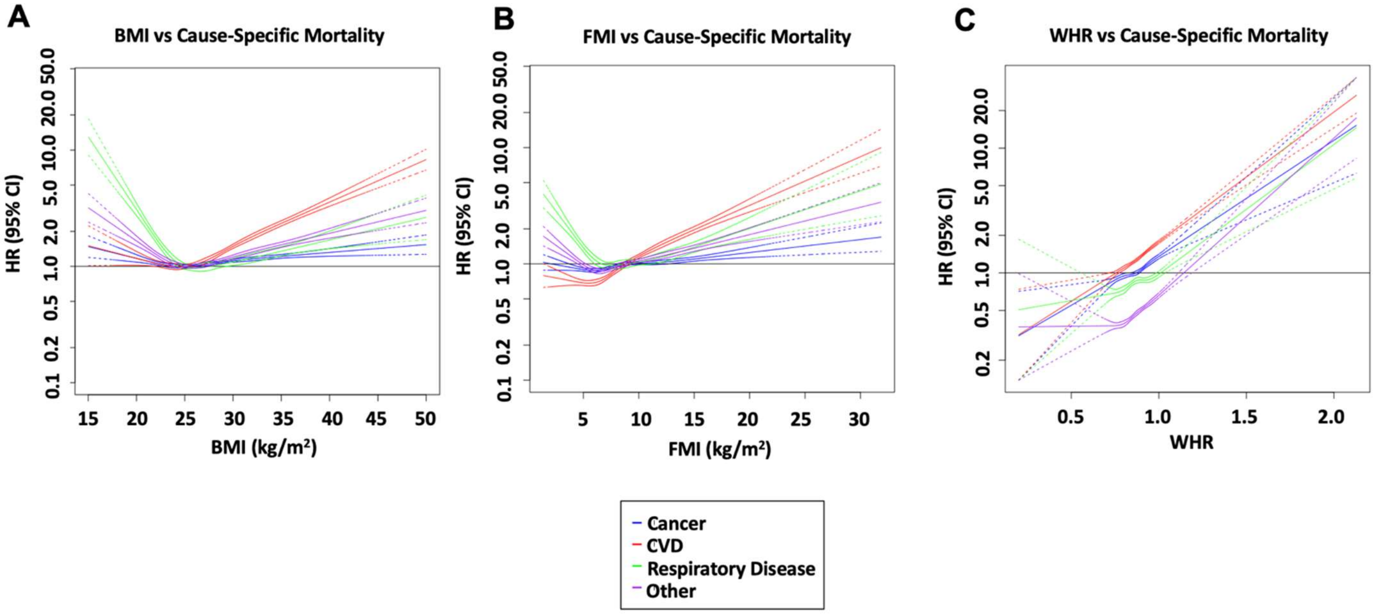

BMI = body mass index, FMI = fat mass index, WHR = Waist-to-hip ratio, CVD = cardiovascular disease, HR = hazard ratio for all-cause mortality. Statistical significance for non-linearity was at a $p < 0.05$. N = 387,672. Against respiratory disease, the nadir for BMI and FMI were 26.0 and 7.43 kg/m^2 respectively. Against other disease, the nadir for BMI and FMI were 25.5 and 6.55 kg/m^2 respectively. [a]Significance set at $p < 0.05$

© 2023 Khan I et al. *JAMA Network Open.*

eTable 6. Linear Mendelian Randomization Analyses Comparing the Association of Individual Genetically Determined Adiposity Measures With All-Cause Mortality Between Premenopausal vs Postmenopausal Females in the UKB.

Stratification by Menopausal Status	OR per SD change in BMI [95% CI]	*P* value	OR per SD change in FMI [95% CI]	*P* value	OR per SD change in WHR [95% CI]	*P* value	*P* value for heterogeneity for differences between groups
**Females of Pre-Menopausal Age**	1.17 [1.00 – 1.36]	0.04 [a]	1.14 [0.98 – 1.34]	0.10	1.22 [0.95 – 1.56]	0.11	0.90
**Females of Post-Menopausal Age**	1.14 [1.08 – 1.22]	1.16×10^{-5} [a]	1.15 [1.08 – 1.22]	9.18×10^{-6} [a]	1.09 [1.00 – 1.19]	0.05	0.63

Pre-menopausal age was defined as females aged 52 and younger, while post-menopausal age was defined as females aged 53 and older. All PRS were standardized for their effects on their corresponding traits (i.e. the BMI PRS was adjusted for its effect on BMI). Odds ratios (OR) indicate the effect of a 1 SD unit increase in adiposity measure on risk of all-cause mortality. Significance is considered at $p < 0.05$. BMI = body mass index, FMI = fat mass index, WHR = waist-to-hip ratio, OR = odds ratio, PRS = polygenic risk score, $P_{het} = p$ value for heterogeneity from the fixed-effects general heterogeneity test. $N_{pre-menopausal}$ = 2,300; $N_{post-menopausal}$ = 18,263. The adjusted model was used. Asterisks (*) represent statistical significance.

© 2023 Khan I et al. *JAMA Network Open.*

eFigure 3. Comparison Between Epidemiologically Derived and MR Derived Estimates for the BMI–All-Cause Mortality Association

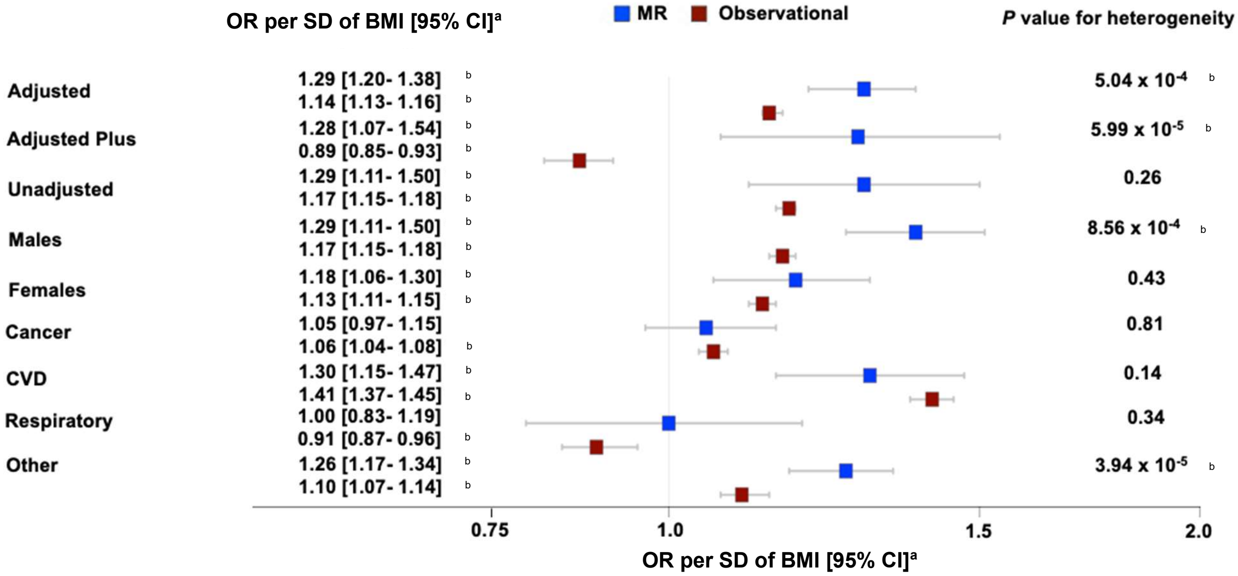

All PRS were standardized for their effects on their corresponding traits (e.g. the BMI PRS was adjusted for its effect on BMI). Hazard ratios (HR) indicate the effect of a 1 SD unit increase in BMI on risk of mortality. Odds ratios (OR) indicate the effect of a 1 SD unit increase in genetically-predicted BMI measure on risk of mortality. The adjusted model was used for all sex-specific and cause-specific mortality analyses. Significance is considered at $p < 0.05$. MR = mendelian randomization, BMI = body mass index, FMI = fat mass index, WHR = waist-to-hip ratio, CVD = cardiovascular disease, OR = odds ratio, HR = hazard ratio, PRS = polygenic risk score. N= 387,672 (adjusted, adjusted plus, unadjusted, and cause-specific mortality models with males and females combined, epidemiological analyses), N=50,594 (adjusted, adjusted plus, unadjusted, and cause-specific mortality models with males and females combined, MR analyses), N=177,340 (males only cohort, epidemiological analyses), N=210,332 (females only cohort, epidemiological analyses), N=30,031 (males only cohort, MR analyses), and N=20,563 (females only cohort, MR analyses). The *p* value for heterogeneity was obtained from the fixed-effects general heterogeneity test. Asterisks (*) represent statistical significance. [a]OR for mendelian randomization analyses, HR for observational analyses. [b] Significance set at $p < 0.05$.

© 2023 Khan I et al. *JAMA Network Open.*

eFigure 4. Comparison Between Epidemiologically Derived and MR-Derived Estimates for the FMI–All-Cause Mortality Association

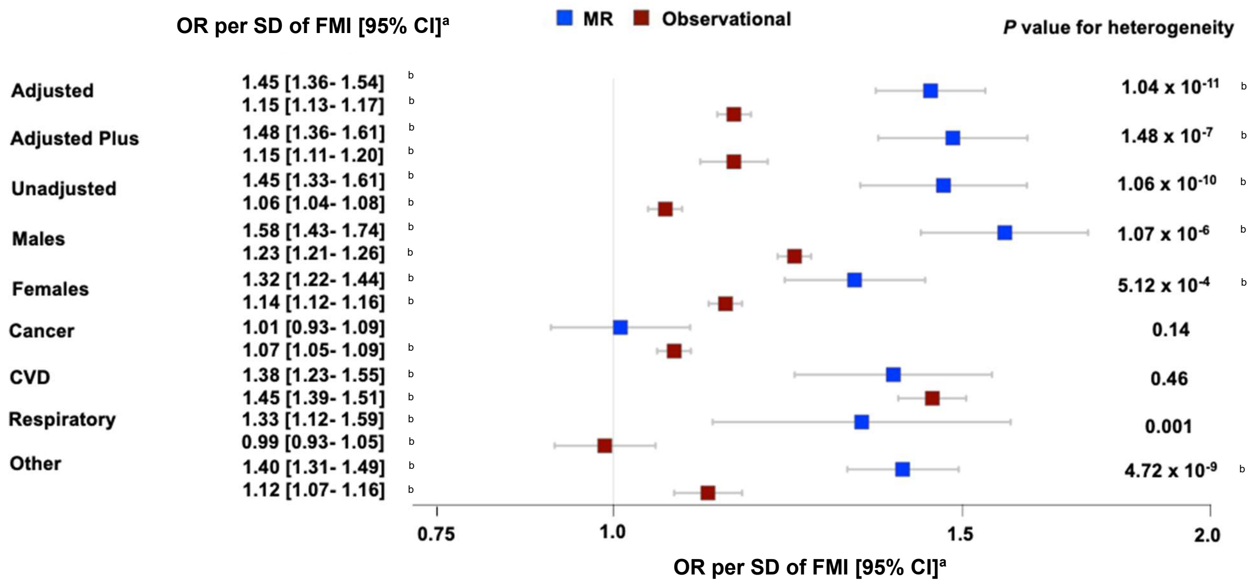

All PRS were standardized for their effects on their corresponding traits (e.g. the FMI PRS was adjusted for its effect on FMI). Hazard ratios (HR) indicate the effect of a 1 SD unit increase in FMI on risk of mortality. Odds ratios (OR) indicate the effect of a 1 SD unit increase in genetically-predicted FMI measure on risk of mortality. The adjusted model was used for all sex-specific and cause-specific mortality analyses. Significance is considered at $p < 0.05$. MR = mendelian randomization, BMI = body mass index, FMI = fat mass index, WHR = waist-to-hip ratio, CVD = cardiovascular disease, OR = odds ratio, HR = hazard ratio, PRS = polygenic risk score. N= 387,672 (adjusted, adjusted plus, unadjusted, and cause-specific mortality models with males and females combined, epidemiological analyses), N=50,594 (adjusted, adjusted plus, unadjusted, and cause-specific mortality models with males and females combined, MR analyses), N=177,340 (males only cohort, epidemiological analyses), N=210,332 (females only cohort, epidemiological analyses), N=30,031 (males only cohort, MR analyses), and N=20,563 (females only cohort, MR analyses). The *p* value for heterogeneity was obtained from the fixed-effects general heterogeneity test. Asterisks (*) represent statistical significance. [a]OR for mendelian randomization analyses, HR for observational analyses. [b] Significance set at $p < 0.05$.

© 2023 Khan I et al. *JAMA Network Open.*

eFigure 5. Comparison Between Epidemiologically Derived and MR-Derived Estimates for the WHR–All-Cause Mortality Association

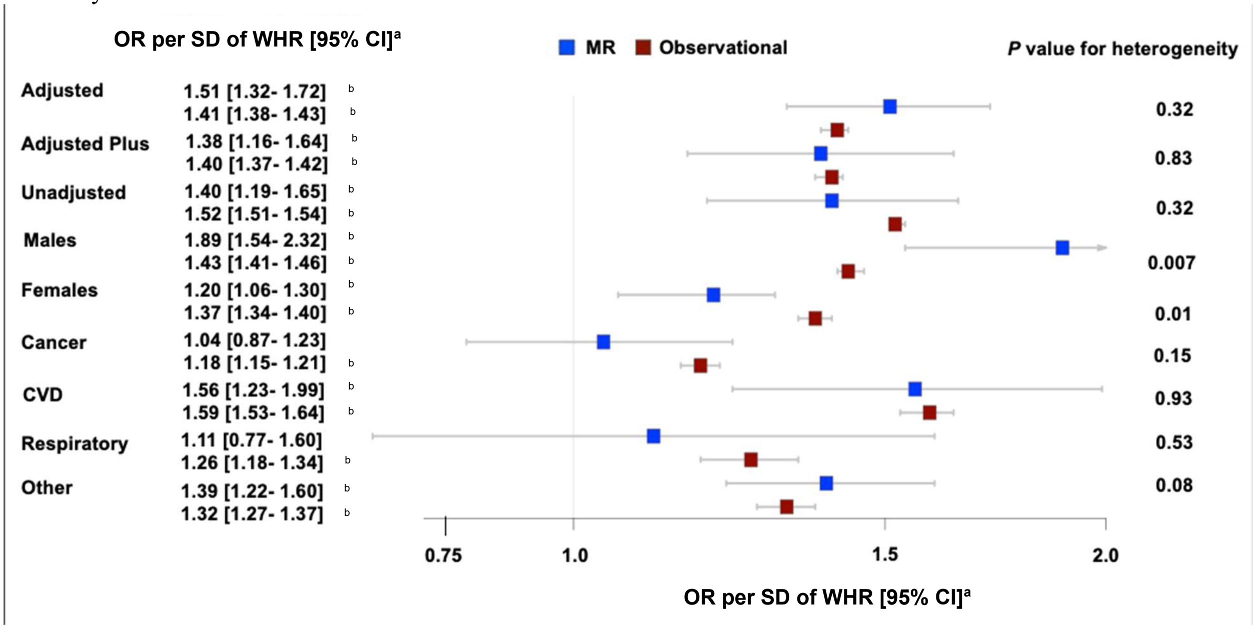

All PRS were standardized for their effects on their corresponding traits (e.g. the WHR PRS was adjusted for its effect on WHR). Hazard ratios (HR) indicate the effect of a 1 SD unit increase in WHR on risk of mortality. Odds ratios (OR) indicate the effect of a 1 SD unit increase in genetically-predicted WHR measure on risk of mortality. The adjusted model was used for all sex-specific and cause-specific mortality analyses. Significance is considered at $p < 0.05$. MR = mendelian randomization, BMI = body mass index, FMI = fat mass index, WHR = waist-to-hip ratio, CVD = cardiovascular disease, OR = odds ratio, HR = hazard ratio, PRS = polygenic risk score. N= 387,672 (adjusted, adjusted plus, unadjusted, and cause-specific mortality models with males and females combined, epidemiological analyses), N=50,594 (adjusted, adjusted plus, unadjusted, and cause-specific mortality models with males and females combined, MR analyses), N=177,340 (males only cohort, epidemiological analyses), N=210,332 (females only cohort, epidemiological analyses), N=30,031 (males only cohort, MR analyses), and N=20,563 (females only cohort, MR analyses). The *p* value for heterogeneity was obtained from the fixed-effects general heterogeneity test. Asterisks (*) represent statistical significance. [a]OR for mendelian randomization analyses, HR for observational analyses. [b] Significance set at $p < 0.05$.

© 2023 Khan I et al. *JAMA Network Open.*

eTable 7. Select Single Nucleotide Variants (SNVs) Associated With BMI

All *p* values were statistically significant at $p < 0.05$.

Chromosome	Base Position	SNPs	Reference Allele	Alternate Allele	*P*-value	Beta	OR
1	136113	rs546872994	T	C	0.008	0.199	1.22
2	22289729	rs328560	G	A	0.005	0.09	1.10
3	104140278	rs9859555	C	A	0.007	-0.03	0.97
4	76551797	rs113618143	A	G	0.002	0.04	1.04
5	78141479	rs12189199	G	T	0.006	-0.04	0.96
6	9130257	rs185818601	A	C	0.004	-0.49	0.61
7	75749694	rs10264760	G	A	0.003	-0.03	0.97
8	54252611	rs141444940	G	A	0.009	-0.06	0.94
9	120891023	rs7866659	G	A	0.004	-0.03	0.97
10	113787569	rs2804591	G	A	0.009	0.04	1.04
11	12054894	rs11022123	A	G	0.005	0.04	1.04
12	18156841	rs10840933	T	C	0.009	-0.04	0.97
13	25141521	rs9551117	G	A	0.004	0.03	1.03
14	34721148	rs17441461	T	C	0.0008	0.04	1.04
15	68989225	rs72625782	T	C	0.003	-0.04	0.96
16	544064	rs143376029	C	T	0.001	0.04	1.04
17	7931282	rs7217076	G	A	0.009	0.07	0.93
18	40808652	rs10502808	G	A	0.0006	0.05	1.05
19	34207555	rs157727	A	G	0.008	0.03	1.03
20	24604300	rs4815290	T	C	0.0002	0.04	1.05
21	31771872	rs553633919	A	G	0.003	0.40	0.67
22	51146439	rs6010060	G	A	0.007	-0.05	0.95

© 2023 Khan I et al. *JAMA Network Open.*

eTable 8. Select Single Nucleotide Variants (SNVs) Associated With FMI

Chromosome	Base Position	SNPs	Reference Allele	Alternate Allele	*P*-value	Beta	OR
1	1545546	rs540146408	G	A	0.005	-0.29	0.75
2	164586665	rs7563112	C	T	0.002	0.03	1.03
3	131580315	rs111740364	T	C	0.008	0.03	1.03
4	97594821	rs7690245	C	T	0.0008	0.03	1.03
5	116911846	rs265917	T	C	0.009	0.02	1.02
6	32615427	rs17843575	T	C	0.0006	-0.03	0.97
7	5501743	rs4640961	C	T	0.009	0.03	1.03
8	71727296	rs13263047	A	G	0.006	-0.02	0.98
9	130302249	rs76589843	C	T	0.005	0.09	1.09
10	1280920	rs547655803	G	T	0.007	-0.43	0.65
11	55923053	rs7938955	T	C	0.005	-0.02	0.98
12	6791077	rs112936609	G	A	0.004	-0.06	0.94
13	72496625	rs191351864	A	C	0.002	0.11	1.11
14	91535660	rs9989151	T	C	0.0009	0.03	1.03
15	72430434	rs4777489	G	A	0.0003	0.03	1.03
16	24773132	rs72768680	A	G	0.005	0.04	1.05
17	41508574	rs34385883	A	G	0.01	-0.02	0.98
18	60674395	rs573747272	C	T	0.007	0.25	1.28
19	9202710	rs79915041	A	G	0.008	-0.073	0.93
20	54389179	rs112226073	T	C	0.0004	0.03	1.03
21	44839654	rs229346	G	A	0.007	-0.03	0.97
22	40585744	rs738450	G	A	0.0002	0.03	1.03

© 2023 Khan I et al. *JAMA Network Open.*

eTable 9. Select Single Nucleotide Variants (SNVs) Associated With WHR

Chromosome	Base Position	SNPs	Reference Allele	Alternate Allele	*P*-value	Beta	OR
1	1310291	rs536418752	A	C	0.004	0.002	1.00
2	524699	rs531034327	A	C	0.002	-0.008	0.99
3	140937223	rs58903469	C	T	0.004	0.0007	1.00
4	55698811	rs2726623	T	C	0.0003	-0.0006	0.99
5	54670639	rs6886473	T	C	0.0002	-0.001	1.00
6	20823756	rs6923264	C	T	0.0001	0.0006	1.00
7	32272429	rs12701194	A	G	0.001	-0.0005	0.99
8	41877174	rs12546563	A	C	0.0007	-0.001	1.00
9	81366872	rs2210417	T	C	0.0002	0.0006	1.00
10	20962310	rs141964776	T	C	0.008	0.002	1.00
11	121922587	rs11218510	A	G	0.0003	0.0006	1.00
12	62055240	rs144906178	A	G	0.0009	-0.003	1.00
13	60779102	rs11617610	T	C	0.0002	0.0006	1.00
14	25942665	rs10143820	T	C	0.0004	0.0006	1.00
15	41055195	rs3759796	C	T	0.005	0.0005	1.00
16	52298333	rs12932031	G	A	0.0008	-0.0007	0.99
17	41141971	rs323496	C	T	0.005	0.0005	1.00
18	48590029	rs2510000	T	C	0.002	-0.001	0.99
19	10074240	rs554922285	T	G	0.0005	0.002	1.00
20	48592000	rs6091075	G	A	0.002	-0.0006	0.99
21	39775084	rs2073359	T	C	0.001	0.0005	1.00
22	51176164	rs76593947	C	T	0.006	-0.002	0.99

© 2023 Khan I et al. *JAMA Network Open.*

Table 10. Egger Regression Mendelian Randomization Analyses

[a] Statistical significance at $p < 0.05$.

BMI

Linkage disequilibrium thresholds for pruning using PLINK:

Clumping window: 500 kb
Clumping R2 threshold: 0.1
Clumping *p* value for significance for index SNPs: 1
Number of SNPs included in the analysis: 389

**Outcome**	**Estimate**	**Estimate (OR)**	***P* value**	***P* value for intercept**
All-cause mortality	0.32	1.32	0.08	0.86
Cancer mortality	-0.15	0.86	0.59	0.14
Cardiovascular disease mortality	0.82	1.82	0.04	0.43
Respiratory disease mortality	0.81	1.81	0.62	0.20
Other disease mortality	0.61	1.61	0.11	0.39

FMI

Linkage disequilibrium thresholds for pruning using PLINK:

Clumping window: 500 kb
Clumping R2 threshold: 0.1
Clumping *p* value for significance for index SNPs: 1
Number of SNPs included in the analysis: 393

**Outcome**	**Estimate**	**Estimate (OR)**	***P* value**	***P* value for intercept**
All-cause mortality	0.06	1.06	0.01[a]	0.13
Cancer mortality	-0.007	0.99	0.85	0.03
Cardiovascular disease mortality	0.09	1.09	0.10	0.31
Respiratory disease mortality	0.12	1.12	0.12	0.13
Other disease mortality	0.08	1.08	0.10	0.90

WHR

Linkage disequilibrium thresholds for pruning using PLINK:

Clumping window: 500 kb
Clumping R2 threshold: 0.1
Clumping *p* value for significance for index SNPs: 1
Number of SNPs included in the analysis: 217

© 2023 Khan I et al. *JAMA Network Open.*

Outcome	Estimate	Estimate (OR)	*P* value	*P* value for intercept
All-cause mortality	-0.03	0.97	0.93	0.34
Cancer mortality	0.17	1.17	0.71	0.85
Cardiovascular disease mortality	0.86	1.86	0.26	0.48
Respiratory disease mortality	0.08	1.08	0.94	0.79
Other disease mortality	-0.26	0.77	0.71	0.40

© 2023 Khan I et al. *JAMA Network Open.*

eTable 11. Inverse Variance Weighted Mendelian Randomization Analyses

BMI

Linkage disequilibrium thresholds for pruning using PLINK:

Clumping window: 500 kb
Clumping R2 threshold: 0.1
Clumping *p* value for significance for index SNPs: 1
Number of SNPs included in the analysis: 389

**Outcome**	**Estimate**	**Estimate (OR)**	***P* value**
All-cause mortality	0.36	1.36	9.35×10^{-13} [a]
Cancer mortality	0.26	1.26	0.0009 [a]
Cardiovascular disease mortality	0.52	1.52	2.37×10^{-6} [a]
Respiratory disease mortality	0.06	1.06	0.72
Other disease mortality	0.30	1.30	0.004 [a]

FMI

Linkage disequilibrium thresholds for pruning using PLINK:

Clumping window: 500 kb
Clumping R2 threshold: 0.1
Clumping *p* value for significance for index SNPs: 1
Number of SNPs included in the analysis: 393

**Outcome**	**Estimate**	**Estimate (OR)**	***P* value**
All-cause mortality	0.09	1.09	2.48×10^{-12} [a]
Cancer mortality	0.06	1.06	0.003 [a]
Cardiovascular disease mortality	0.13	1.13	5.76×10^{-6} [a]
Respiratory disease mortality	0.02	1.02	0.61
Other disease mortality	0.08	1.08	0.005 [a]

WHR

Linkage disequilibrium thresholds for pruning using PLINK:

Clumping window: 500 kb
Clumping R2 threshold: 0.1
Clumping *p* value for significance for index SNPs: 1
Number of SNPs included in the analysis: 217

**Outcome**	**Estimate**	**Estimate (OR)**	***P* value**
All-cause mortality	0.29	1.29	6.72×10^{-5} [a]
Cancer mortality	0.26	1.26	0.008 [a]
Cardiovascular disease mortality	0.33	1.33	0.04 [a]

© 2023 Khan I et al. *JAMA Network Open.*

Respiratory disease mortality	0.36	1.36	0.11
Other disease mortality	0.32	1.34	0.03 [a]

© 2023 Khan I et al. *JAMA Network Open.*

eTable 12. Weighted Median Mendelian Randomization Analyses

BMI

Linkage disequilibrium thresholds for pruning using PLINK:

Clumping window: 500 kb
Clumping R2 threshold: 0.1
Clumping *p* value for significance for index SNPs: 1
Number of SNPs included in the analysis: 389

**Outcome**	**Estimate**	**Estimate (OR)**	***P* value**
All-cause mortality	0.31	1.31	3.68×10^{-5} [a]
Cancer mortality	0.17	1.17	0.13
Cardiovascular disease mortality	0.48	1.48	0.004 [a]
Respiratory disease mortality	0.06	1.06	0.82
Other disease mortality	0.15	1.15	0.38

FMI

Linkage disequilibrium thresholds for pruning using PLINK:

Clumping window: 500 kb
Clumping R2 threshold: 0.1
Clumping *p* value for significance for index SNPs: 1
Number of SNPs included in the analysis: 393

**Outcome**	**Estimate**	**Estimate (OR)**	***P* value**
All-cause mortality	1.07	1.07	0.002 [a]
Cancer mortality	-0.01	0.99	0.72
Cardiovascular disease mortality	0.13	1.13	0.02 [a]
Respiratory disease mortality	0.11	1.11	0.15
Other disease mortality	0.04	1.04	0.41

WHR

Linkage disequilibrium thresholds for pruning using PLINK:

Clumping window: 500 kb
Clumping R2 threshold: 0.1
Clumping *p* value for significance for index SNPs: 1
Number of SNPs included in the analysis: 217

**Outcome**	**Estimate**	**Estimate (OR)**	***P* value**
All-cause mortality	0.30	1.30	0.002 [a]
Cancer mortality	0.17	1.17	0.20
Cardiovascular disease mortality	0.26	1.26	0.24

© 2023 Khan I et al. *JAMA Network Open.*

Respiratory disease mortality	0.41	1.41	0.22
Other disease mortality	0.34	1.34	0.10

eTable 13. Summary of Epidemiological and Linear Mendelian Randomization Analyses of Adiposity Measures and All-Cause Mortality: Full Adjusted Model

Hazard ratios (HR) indicate the effect of a 1 SD unit increase in adiposity measure on risk of all-cause mortality. Odds ratios (OR) indicate the effect of a 1 SD unit increase in genetically-determined adiposity measure on risk of all-cause mortality. The adjusted model was used for all sex-specific analyses. MR = mendelian randomization, BMI = body mass index, FMI = fat mass index, WHR = waist-to-hip ratio, OR = odds ratio, HR = hazard ratio, PRS = polygenic risk score. N=50,594 (adjusted model with males and females combined), N=30,031 (males only cohort), and N=20,563 (females only cohort). All relationships have $p < 0.05$.

All-Cause Mortality		OR/HR per SD change in BMI [95% CI]	OR/HR per SD change in FMI [95% CI]	OR/HR per SD change in WHR [95% CI]
**Fully adjusted model**	MR	1.14 [1.04 - 1.25]	1.24 [1.13 - 1.36]	1.13 [0.92 - 1.40]
	Epidemiological	0.87 [0.82 - 0.90]	1.15 [1.09 - 1.22]	1.30 [1.27 - 1.33]

© 2023 Khan I et al. *JAMA Network Open.*

eFigure 6. Nonlinear Mendelian Randomization Analyses Comparing Genetically Determined Adiposity Measure–All-Cause Mortality Associations Across BMI Quantiles

All PRS were standardized for their effects on their corresponding traits (i.e. the BMI PRS was adjusted for its effect on BMI). Significance is considered at $p < 0.05$. Asterisks (*) represent statistical significance. Statistical significance indicates inconsistency of the adiposity-mortality relationship across quantiles of the relevant adiposity measure. The horizontal line indicates an OR of 1. BMI = body mass index, FMI = fat mass index, WHR = waist-to-hip ratio, PRS = polygenic risk score, OR= odds ratio for all-cause mortality per SD of the adiposity measure, $P_{\text{non-linearity}}$ = p value from ANOVA testing for non-linearity. n = 50,007.

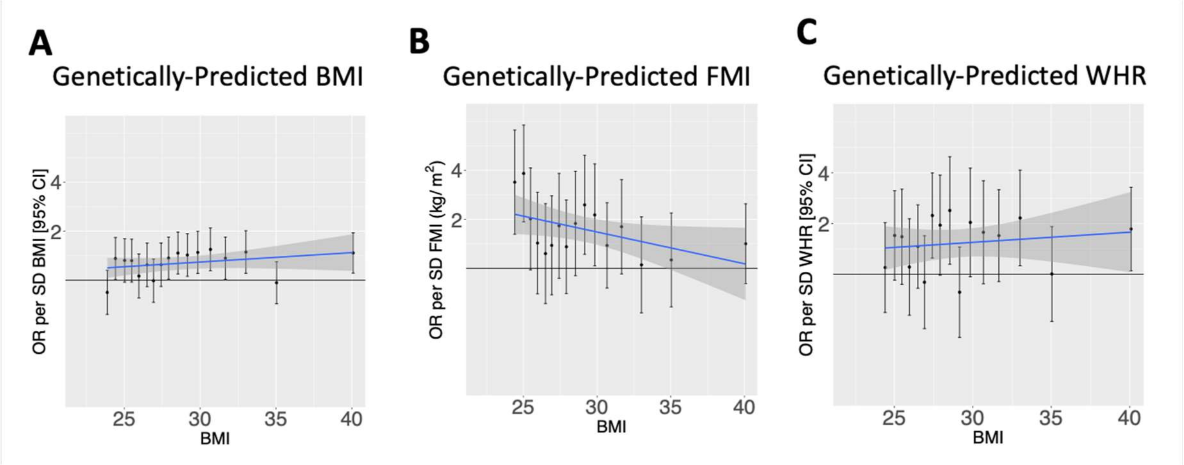

© 2023 Khan I et al. *JAMA Network Open.*

eFigure 7. Epidemiological Association Between BMI, FMI, and WHR and All-Cause Mortality Across Age Groups

The relationship between a) BMI, b) FMI, and c) WHR respectively across different age groups. BMI = body mass index, FMI = fat mass index, WHR = Waist-to-hip ratio, HR = hazard ratio for all-cause mortality. Statistical significance for non-linearity was defined as $p < 0.05$. $N_{under\ 49} = 74,003$, $N_{49\text{-}56} = 80,934$, $N_{56\text{-}61} = 77,109$, $N_{61\text{-}64} = 60,329$ and $N_{over\ 64} = 95,269$. Across age groups <49, 49-56, 56-61, 61-64, and >64, the nadir for BMI were 26.1, 24.8, 25.5, 25.1 and 25.0 kg/m^2 respectively. Across age groups <49, 49-56, 56-61, 61-64, and >64, the nadir for FMI were 5.42, 6.11, 6.55, 6.31, and 6.30 kg/m^2 respectively.

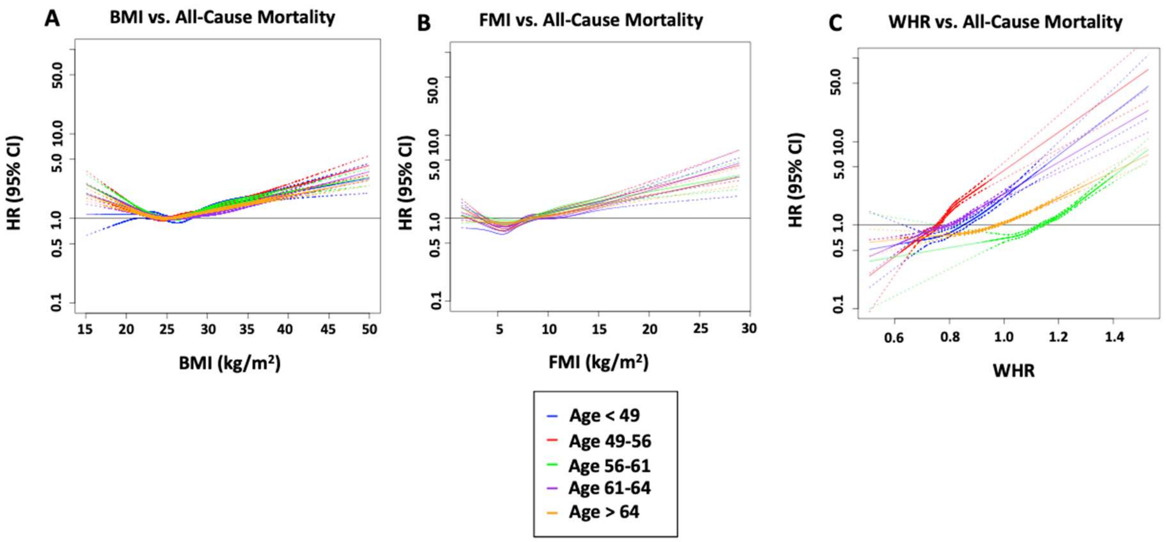

© 2023 Khan I et al. *JAMA Network Open.*

eTable 14. Summary of Epidemiological and Linear Mendelian Randomization Analyses of Adiposity Measures and All-Cause Mortality Across Age Groups

All PRS were standardized for their effects on their corresponding traits (i.e. the BMI PRS was adjusted for its effect on BMI). Odds ratios (OR) indicate the effect of a 1 SD unit increase in adiposity measure on risk of all-cause mortality. Significance is considered at $p < 0.05$. BMI = body mass index, FMI = fat mass index, WHR = waist-to-hip ratio, OR = odds ratio, PRS = polygenic risk score, Observational analyses: $N_{under\ 49} = 74{,}003$, $N_{49\text{-}56} = 80{,}934$, $N_{56\text{-}61} = 77{,}109$, $N_{61\text{-}64} = 60{,}329$, and $N_{over\ 64} = 95{,}269$. MR analyses: $N_{under\ 49} = 2{,}604$, $N_{49\text{-}56} = 5{,}428$, $N_{56\text{-}61} = 9{,}080$, $N_{61\text{-}64} = 10{,}056$ and $N_{over\ 64} = 23{,}426$. The adjusted model was used. [a]OR for mendelian randomization analyses, HR for observational analyses. [b]Statistical significance at $p < 0.05$.

All-Cause Mortality		OR per SD change in BMI [95% CI][a]	*P* value	OR per SD change in FMI [95% CI][a]	*P* value	OR per SD change in WHR [95% CI][a]	*P* value
**Age < 49**	MR	1.01 [0.77 – 1.32]	0.96	1.22 [0.94 – 1.59]	0.14	1.06 [0.62 – 1.81]	0.82
	Epidemiological	1.17 [1.12 – 1.23]	<0.001[b]	1.22 [1.16 – 1.29]	<0.001[b]	1.57 [1.46 – 1.68]	<0.001[b]
**Age 49-56**	MR	1.40 [1.17 – 1.67]		1.89 [1.58 – 2.26]		2.44 [1.68 – 3.53]	
	Epidemiological	1.19 [1.15 – 1.23]		1.23 [1.19 – 1.28]		1.43 [1.39 – 1.48]	
**Age 56-61**	MR	1.34 [1.16 – 1.55]		1.62 [1.40 – 1.88]		1.29 [0.93 – 1.81]	0.13
	Epidemiological	1.15 [1.12 – 1.19]		1.19 [1.15 – 1.23]		1.49 [1.44 – 1.55]	<0.001[b]
**Age 61-64**	MR	1.27 [1.10 – 1.47]	0.0014[b]	1.37 [1.18 – 1.57]	<0.001[b]	1.36 [1.06 – 1.76]	0.02[b]
	Epidemiological	1.14 [1.11 – 1.17]	<0.001[b]	1.17 [1.13 – 1.20]	<0.001[b]	1.42 [1.37 – 1.47]	<0.001[b]
**Age > 64**	MR	1.28 [1.15 – 1.42]	<0.001[b]	1.36 [1.23 – 1.50]	<0.001[b]	1.53 [1.24 – 1.89]	<0.001[b]
	Epidemiological	1.14 [1.12 – 1.16]	<0.001[b]	1.16 [1.14 – 1.19]	<0.001[b]	1.37[1.34 – 1.40]	<0.001[b]

© 2023 Khan I et al. *JAMA Network Open.*

eFigure 8. Comparison Between Epidemiologically Derived and MR-Derived Estimates for the BMI–All-Cause Mortality Association Between Age Groups

All PRS were standardized for their effects on their corresponding traits (e.g. the BMI PRS was adjusted for its effect on BMI). Hazard ratios (HR) indicate the effect of a 1 SD unit increase in BMI on risk of mortality. Odds ratios (OR) indicate the effect of a 1 SD unit increase in genetically-predicted BMI measure on risk of mortality. The adjusted model was used for all age-stratified analyses. Significance is considered at $p < 0.05$. MR = mendelian randomization, BMI = body mass index, OR = odds ratio, HR = hazard ratio, PRS = polygenic risk score. Observational analyses: $N_{under\ 49} = 74{,}003$, $N_{49\text{-}56} = 80{,}934$, $N_{56\text{-}61} = 77{,}109$, $N_{61\text{-}64} = 60{,}329$, and $N_{over\ 64} = 95{,}269$. MR analyses: $N_{under\ 49} = 2{,}604$, $N_{49\text{-}56} = 5{,}428$, $N_{56\text{-}61} = 9{,}080$, $N_{61\text{-}64} = 10{,}056$ and $N_{over\ 64} = 23{,}426$. The p value for heterogeneity was obtained from the fixed-effects general heterogeneity test. Asterisks (*) represent statistical significance. [a]OR for mendelian randomization analyses, HR for observational analyses. [b]Statistical significance at $p < 0.05$.

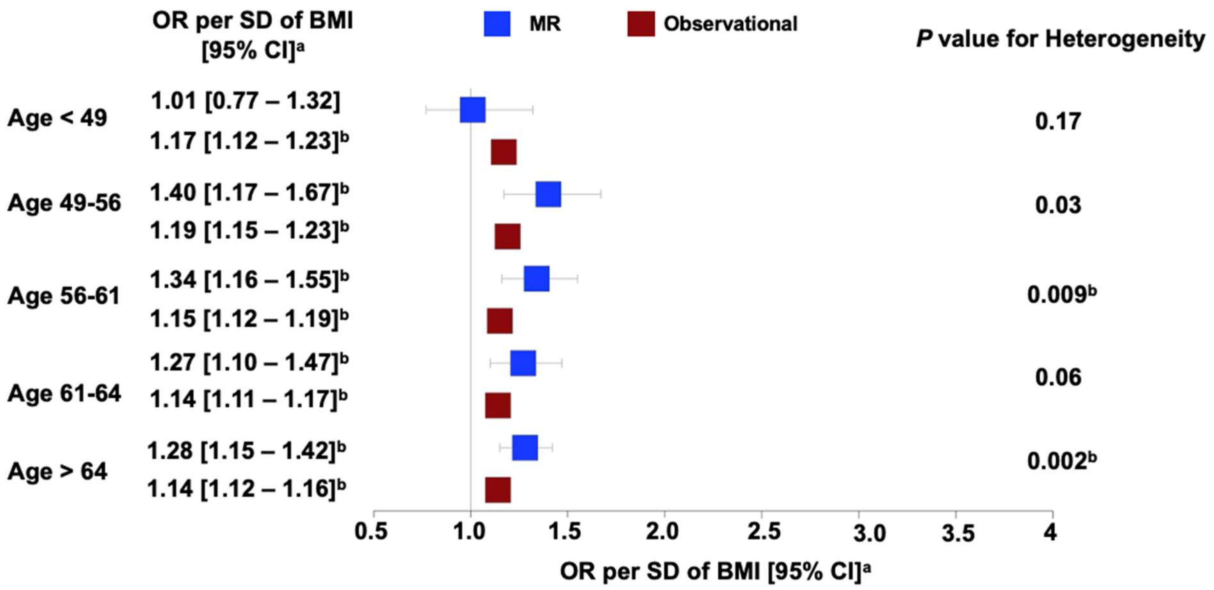

© 2023 Khan I et al. *JAMA Network Open.*

eFigure 9. Comparison Between Epidemiologically Derived and MR-Derived Estimates for the FMI–All-Cause Mortality Association Between Age Groups

All PRS were standardized for their effects on their corresponding traits (e.g. the FMI PRS was adjusted for its effect on FMI). Hazard ratios (HR) indicate the effect of a 1 SD unit increase in FMI on risk of mortality. Odds ratios (OR) indicate the effect of a 1 SD unit increase in genetically-predicted FMI measure on risk of mortality. The adjusted model was used for all age-stratified analyses. Significance is considered at $p < 0.05$. MR = mendelian randomization, FMI = fat mass index, OR = odds ratio, HR = hazard ratio, PRS = polygenic risk score. Observational analyses: $N_{under\ 49} = 74{,}003$, $N_{49\text{-}56} = 80{,}934$, $N_{56\text{-}61} = 77{,}109$, $N_{61\text{-}64} = 60{,}329$, and $N_{over\ 64} = 95{,}269$. MR analyses: $N_{under\ 49} = 2{,}604$, $N_{49\text{-}56} = 5{,}428$, $N_{56\text{-}61} = 9{,}080$, $N_{61\text{-}64} = 10{,}056$ and $N_{over\ 64} = 23{,}426$. The p value for heterogeneity was obtained from the fixed-effects general heterogeneity test. Asterisks (*) represent statistical significance. [a]OR for mendelian randomization analyses, HR for observational analyses. [b]Statistical significance at $p < 0.05$.

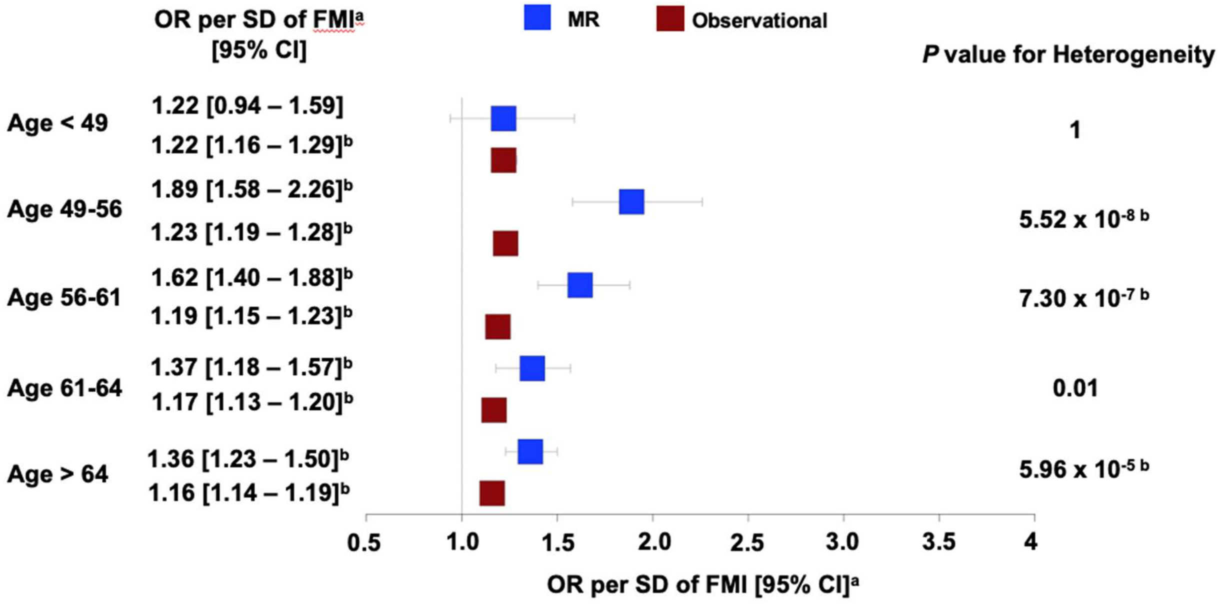

© 2023 Khan I et al. *JAMA Network Open.*

eFigure 10. Comparison Between Epidemiologically Derived and MR-Derived Estimates for the WHR–All-Cause Mortality Association Between Age Groups

Comparison between epidemiologically-derived and MR-derived estimates for the WHR – all-cause mortality relationship between age groups. All PRS were standardized for their effects on their corresponding traits (e.g. the WHR PRS was adjusted for its effect on WHR). Hazard ratios (HR) indicate the effect of a 1 SD unit increase in WHR on risk of mortality. Odds ratios (OR) indicate the effect of a 1 SD unit increase in genetically-predicted WHR measure on risk of mortality. The adjusted model was used for all age-stratified analyses. Significance is considered at $p < 0.05$. MR = mendelian randomization, WHR = waist-to-hip ratio, OR = odds ratio, HR = hazard ratio, PRS = polygenic risk score. Observational analyses: $N_{under\ 49} = 74{,}003$, $N_{49\text{-}56} = 80{,}934$, $N_{56\text{-}61} = 77{,}109$, $N_{61\text{-}64} = 60{,}329$, and $N_{over\ 64} = 95{,}269$. MR analyses: $N_{under\ 49} = 2{,}604$, $N_{49\text{-}56} = 5{,}428$, $N_{56\text{-}61} = 9{,}080$, $N_{61\text{-}64} = 10{,}056$ and $N_{over\ 64} = 23{,}426$. The p value for heterogeneity was obtained from the fixed-effects general heterogeneity test. Asterisks (*) represent statistical significance. [a]OR for mendelian randomization analyses, HR for observational analyses. [b]Statistical significance at $p < 0.05$.

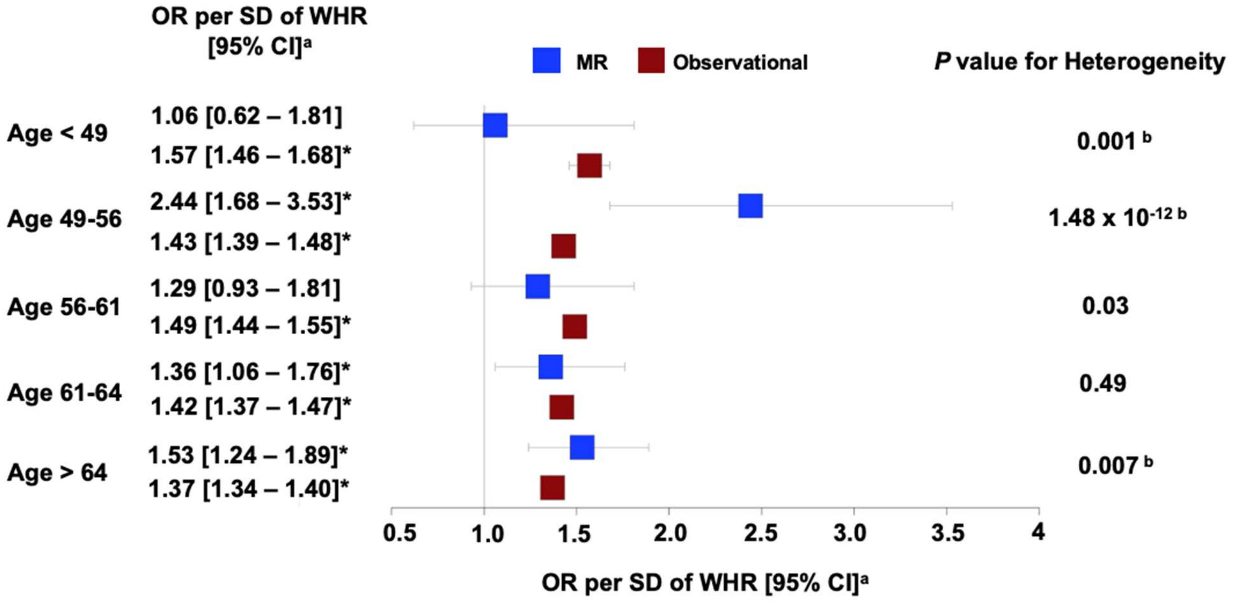

© 2023 Khan I et al. *JAMA Network Open.*

eTable 15. Comparison Between the Cox Regression and Logistic Regression Model in the Adjusted Linear Mendelian Randomization Analysis

The adjusted model was used for all analyses. MR = mendelian randomization, BMI = body mass index, FMI = fat mass index, WHR = waist-to-hip ratio, OR = odds ratio, HR = hazard ratio, PRS = polygenic risk score. N=50,594, All relationships have $p < 0.05$.

All-Cause Mortality	BMI	FMI	WHR
**Logistic Regression (OR per SD change (95% CI))**	1.29 (1.20 – 1.38)	1.45 (1.36 – 1.54)	1.51 (1.32 – 1.72)
**Cox Proportional Hazards Regression (HR per SD change (95% CI))**	1.14 (1.10 – 1.20)	1.21 (1.15 - 1.26)	1.30 (1.18 – 1.42)

© 2023 Khan I et al. *JAMA Network Open.*

eTable 16. Comparison Between the Cox Regression and Logistic Regression Model in the Adjusted Observational Analysis

The adjusted model was used for all analyses. MR = mendelian randomization, BMI = body mass index, FMI = fat mass index, WHR = waist-to-hip ratio, OR = odds ratio, HR = hazard ratio, PRS = polygenic risk score. N=387,672. All relationships have $p < 0.05$.

**All-Cause Mortality**	**BMI**	**FMI**	**WHR**
**Logistic Regression (OR per SD change (95% CI))**	1.15 (1.14 – 1.17)	1.19 (1.17 – 1.21)	1.38 (1.36 – 1.40)
**Cox Proportional Hazards Regression (HR per SD change (95% CI))**	1.06 (1.05 – 1.08)	1.14 (1.13 - 1.16)	1.22 (1.20 – 1.24)

© 2023 Khan I et al. *JAMA Network Open.*

eReferences

1. Locke AE, Kahali B, Berndt SI, *et al*. Genetic studies of body mass index yield new insights for obesity biology. *Nature* 2015; **518**: 197-206. DOI: 10.1038/nature14177.
2. The CARDIoGRAMplusC4D Consortium. Large-scale association analysis identifies new risk loci for coronary artery disease. *Nat Genet* 2013;**45**: 25–33. DOI: 10.1038/ng.2480.
3. Lagou V, Mägi R, Hottenga JJ. *et al*. Sex-dimorphic genetic effects and novel loci for fasting glucose and insulin variability. *Nat Commun* 2021;**12**, 1-18. DOI: 10.1038/s41467-020-19366-9
4. Pigeyre M, Sjaarda J, Mao S, *et al*. Identification of novel causal blood biomarkers linking metabolically favorable adiposity with type 2 diabetes risk. *Diabetes Care* 2019; **42**: 1800-1808. DOI: 10.2337/dc18-2444.
5. Klarin D, Damrauer SM, Cho K, *et al.* Genetics of blood lipids among ~300,000 multi-ethnic participants of the Million Veteran Program. Nat Genet, 2018; **50**, 1514-1523. DOI: 0.1038/s41588-018-0222-9.
6. Medina-Gomez C, Kemp JP, Dimou NL, *et al*. Bivariate genome-wide association meta-analysis of pediatric musculoskeletal traits reveals pleiotropic effects at the SREBF1/TOM1L2 locus. *Nat Commun* 2017;**8**:121. DOI: 10.1038/s41467-017-00108-3.
7. Jung K, Lee J, Gupta V, *et al*. Comparison of Bootstrap Confidence Interval Methods for GSCA Using a Monte Carlo Simulation. *Front Psychol,* 2019; **10**: 2215. doi: 10.3389/fpsyg.2019.02215.
8. Bradbury KE, Guo W, Cairns BJ, *et al.* Association between physical activity and body fat percentage, with adjustment for BMI: a large cross-sectional analysis of UK Biobank. *BMJ Open* 2017;**7**:e011843. DOI: 10.1136/bmjopen-2016-011843.
9. Mbatchou J, Barnard L, Backman J, *et al.* Computationally efficient whole-genome regression for quantitative and binary traits. *Nat Genet.* 2021;**53**:1097-1103. DOI:10.1038/s41588-021-00870-7
10. Sun YQ, Burgess S, Staley JR, *et al*. Body mass index and all cause mortality in HUNT and UK Biobank studies: linear and non- linear mendelian randomisation analyses. *BMJ* 2019, **364**, 1-10. DOI: 10.1136/bmj.l1042.
11. Bhaskaran K, Dos-Santos-Silva I, Leon DA, et al. Association of BMI with overall and cause-specific mortality: a population-based cohort study of 3.6 million adults in the UK. *Lancet Diabetes Endocrinol* 2018; **6**: 944-953. DOI: 10.1016/S2213-8587(18)30288-2.
12. Staley JR & Burgess S. Semiparametric methods for estimation of a nonlinear exposure-outcome relationship using instrumental variables with application to Mendelian randomization. *Genet Epidemiol.* 2017, **41**, 341-352. DOI: 10.1002/gepi.22041.
13. Yengo L, Sidorenko J, Kemper KE, *et al*. Meta-analysis of genome-wide association studies for height and body mass index in ~700,000 individuals of European ancestry. Biorxiv 2018. Retrieved from: https://portals.broadinstitute.org/collaboration/giant/index.php/GIANT_consortium_data_files#BMI_and_Height_GIANT_and_UK_BioBank_Meta-analysis_Summary_Statistics
14. Paré G, Mao S, Deng WQ. A robust method to estimate regional polygenic correlation under misspecified linkage disequilibrium structure. *Genet Epidemiol* 2019; **42**: 636-647. DOI: 10.1002/gepi.22149.
15. Marees AT, de Kluiver H, Stringer S, *et al.* A tutorial on conducting genome-wide association studies: Quality control and statistical analysis. *Int J Methods Psychiatr Res* 2018; **27**: 1-10. DOI: 10.1002/mpr.1608.
16. Guo W, Key TJ, Reeves GK. Adiposity and breast cancer risk in postmenopausal women: Results from the UK Biobank prospective cohort. *Int J Cancer.* 2018;**143**(5):1037-1046. doi:10.1002/ijc.31394
17. Hemani G, Zheng J, Elsworth B *et al.* The MR-Base platform supports systematic causal inference across the human phenome. *eLife* 2018;**7**:e34408. doi: 10.7554/eLife.34408.

© 2023 Khan I et al. *JAMA Network Open.*
