# Supplementary material for: Surrogate Adiposity Markers and Mortality
Source: JAMA Netw Open. 2023 Sep 20;6(9):e2334836. doi: 10.1001/jamanetworkopen.2023.34836 (PMC10512100; doi:10.1001/jamanetworkopen.2023.34836)
Supplement: Supplement 2. — Data Sharing Statement [file jamanetwopen-e2334836-s002.pdf]

Data Sharing Statement

Khan. Surrogate Adiposity Markers and Mortality Among White Adults. *JAMA Netw Open*. Published September 20, 2023. doi:10.1001/jamanetworkopen.2023.34836

Data
Data available: Yes
Data types: Deidentified participant data
How to access data: Please find the link to the UK Biobank Data Showcase here: https://biobank.ndph.ox.ac.uk/showcase/ Email address to request access: access@ukbiobank.ac.uk
When available: With publication

Supporting Documents
Document types: None

Additional Information
Who can access the data: Data will be made available for anyone requesting the data through the UK Biobank application process.
Types of analyses: Data will be made available for anyone requesting the data through the UK Biobank application process.
Mechanisms of data availability: Data will be made available for anyone requesting the data through the UK Biobank application process.
Any additional restrictions: All UK Biobank data from our study can be accessed through an application process that must be completed on the main UK Biobank website.
